# Use of Different Types of Biosorbents to Remove Cr (VI) from Aqueous Solution

**DOI:** 10.3390/life11030240

**Published:** 2021-03-14

**Authors:** Eva Pertile, Tomáš Dvorský, Vojtěch Václavík, Silvie Heviánková

**Affiliations:** Department of Environmental Engineering, Faculty of Mining and Geology, VSB—Technical University of Ostrava, 708 00 Ostrava, Czech Republic; vojtech.vaclavik@vsb.cz (V.V.); silvie.heviankova@vsb.cz (S.H.)

**Keywords:** biosorption, hexavalent chromium Cr (VI), batch mode, kinetic, equilibrium, thermodynamic study

## Abstract

This article summarizes the results of a research study that was focused on the possibility of removing Cr (VI) from aqueous solution, using low-cost waste biomaterial in a batch mode. A set of seven biosorbents was used: *Fomitopsis pinicola*, a mixture of cones, peach stones, apricot stones, *Juglans regia* shells, orange peels, and Merino sheep wool. Three grain fractions (fr. 1/2, fr. 0.5/1.0, and fr. 0/0.5 mm) of biosorbents were studied. The aim was to find the most suitable biosorbent that can be tested with real samples. The influence of other factors on the course of biosorption was studied as well (chemical activation of the biosorbent, pH value, rotation speed during mixing, temperature, and the influence of biosorbent concentration). The use of chemical activation and adjustment of the pH to 1.1 to 2.0 make it possible to increase their sorption capacity and, for some biosorbents, to shorten the exposure times. Two kinetic models were used for the analysis of the experimental data, to explain the mechanism of adsorption and its possible speed control steps: pseudo-first and pseudo-second-order. The pseudo-second-order kinetic model seems to be the most suitable for the description of the experimental data. The thermodynamic parameters suggest that the biosorption was endothermic and spontaneous. In the biosorption equilibrium study, the adsorption data were described by using Langmuir and Freundlich adsorption isotherms. The Langmuir model was applicable to describe the adsorption data of all biosorbents. Both models are suitable for chemically treated sheep fleece and peach stones.

## 1. Introduction

Although water is priceless, from the point of view of the life of organisms and the landscape, it is too often underestimated by humans. Nowadays, we have virtually the same sources of drinking water as people had hundreds of years ago. The difference, however, is that there are more than seven billion people currently sharing the same resources. The planning, implementation, and use of wastewater treatment systems are therefore more important now than in the past. The issue of heavy metals, with regard to their toxicity, persistence, and bioaccumulation capacity in the environment, still attracts a lot of attention. Their presence in industrial wastewater is often a major problem with respect to their discharge into surface water. Chromium, which is used in a number of industrial applications, can be included among these problematic metals as well. Wastewater contains both hexavalent and trivalent chromium in a concentration 10–100 mg·L^−1^. A wide variety of physical and chemical processes can be used to remove hexavalent chromium from the aqueous environment. However, effective, simple, and, most importantly, inexpensive methods for removing toxic metals have recently been sought. Our attention is more and more often focused on the use of sorption properties of various low-cost, abundant materials. The interesting properties of these sorbents include their high versatility, relatively good metal selectivity, and, in some cases, high sorption capacity, especially in the low range of concentrations. On the other hand, it should be noted that not all of these naturally available materials that have been studied so far have had a satisfactory sorption capacity in the verified concentration range.

For economic reasons, agricultural waste, which can be considered as low-cost biosorbents, is also in the center of attention [1]. Although many biological materials can bind heavy metals, only those that have sufficiently high selectivity and the ability to bind heavy metals are suitable for full-scale use in the biosorption process. Untreated crude biosorbents usually have a lower sorption capacity for metal ions than, for example, chemically modified biosorbents, because their surface lacks suitable chemical functional groups. That is why various pre-treatments can be used to increase the efficiency of metal ion removal by means of biosorbents. Sorbent pre-treatment may involve, for example, strengthening the cell wall structure by crosslinking, using epichlorohydrin [2]; increasing the negative charge on the cell surface, by alkali treatment (most often NaOH) [3]; or increasing the positive charge on the cell surface by acid treatment [4,5], thus opening the potential adsorption sites.

A number of materials have already been studied as potential biosorbents to remove Cr (VI) as well. The main components of the waste material studied by us include cellulose, hemicellulose, and lignin. This type of waste is also classified as lignocellulosic waste. In most cases, these studied lignocellulosic biosorbents are further modified by using various chemical methods to increase the sorption capacity of the metal [6]. Many studies have shown that metal bonding occurs especially through a chemical functional group (carboxyl and hydroxyl groups).

Although orange peels (*Citrus sinensis*) do not belong to the traditional assortment in our geographical latitudes, they have been selected from the set of the tested biosorbents due to their composition (high pectin content). Previous research has been focused mainly on the removal of the following metals: Ni [7,8,9], Pb [7,8,9,10,11,12,13,14], As, Cu, Cd [7,8,11], Cu [11,13,15,16,17,18,19], Fe (III), Cr (III) [20,21], Cr (VI) [22,23,24], Zn (II) [13,14] Co and Mo [9], and As [25,26]. The information on the structure and properties of orange peels was taken from the professional literature cited above, because the presented findings were practically identical in all works. The use of orange peels as a potential adsorbent material provides a great potential, especially due to their high content of cellulose, pectin, hemicellulose, and lignin. These components contain polar functional groups (carboxyl and phenolic), which may also be involved in the bond. Perez, Lugo-Lugo et al. [8,17,20,21,27,28] state that the IR spectra show a number of absorption peaks, suggesting the complex nature of the material studied. Since orange peel is mostly composed of cellulose, pectic acid, and pectin, O–H, C–O, C=O, C–H, and C–C bonds are expected.

A number of works dealing with the ability of shells of various types of fruit (hazelnuts [29,30,31], almonds [30,32,33,34], walnuts [35,36,37,38,39,40], groundnut [41,42,43,44], pistachios [30,45,46], coconuts [47,48,49,50,51], etc.) to sorb metal ions from the aqueous environment can also be found in the literature sources. It is cheap, easily available agricultural waste biomass. Previous research in the field of their possible use for biosorption has focused mainly on the removal of the following metals: Cd, Zn, Cr (III), Cr (VI), Cs^+^, As, and Pb [29,35,36]. Information on the structure and properties of walnut shells (*Juglans regia*) has also been taken from the reference sources mentioned above.

Unmodified walnut shells (*Juglans regia*) have a relatively complex and multilayer fibrous lignocellulosic structure. Their surface is rough, with a large number of pores, which represent the possible sites for Cr (VI) biosorption by means of physical or chemical mechanism. The main components of walnut shells are cellulose, hemicellulose and lignin, which is the predominant structural component (40.7–48.6%). They also contain some other polar functional groups, such as alcohol, carbonyl, carboxyl, and phenolic ones [29,35,36,52,53]. 

The common occurrence of conifers in the Czech Republic is related to the abundant source of unused cone biomass as a renewable resource. The biomass of conifer fruits is itself forest waste, and it is essentially a readily available potential biosorbent. The ripe cone consists of epidermis and sclerenchymatic cells, which contain cellulose, hemicellulose, and lignin in their cell walls [54]. Cone biomass composed of polysaccharides can thus provide binding amino, carboxyl, phosphate, and sulfate groups to the metal–biosorbent bond [55]. A wide range of different types of cones have been tested in the professional literature dealing with biosorption for various metals, such as Pb [56], Cu [54,57,58,59,60], Zn [61], Cr (VI) [54,62], Ni (II) [63,64], and Cd (II) [56,57,65,66]. 

Apricots (*Prunus armeniaca*) and peaches (*Prunus persica*), as seasonal fruit, are widely consumed in our geographical latitude. They can also be a serious environmental problem as an agricultural by-product. Apricot and peach stones are therefore an inexpensive and widely available material that can be further processed for the purpose of biosorption. In the professional literature dealing with biosorption, apricot and peach stones have already been studied for the adsorption of, for example, Pb [67], Cd [30], Zn [30], Cu [30,68,69], and Cr (VI) [70]. An analysis of the chemical composition has shown that the main components of stones are, again, cellulose (30% of weight) and hemicellulose (28% of weight), lignin (30% of weight), and small amounts of lipids (12% of weight) [30,71].

Wood-decaying fungus *Fomitopsis pinicola* has been tested to study the biosorption of hexavalent chromium from the aqueous environment. The cell walls of fungi consist of 80–90% of heteropolysaccharides, proteins, lipids, polyphosphates, and inorganic ions, which form the walls, using sealant mass [72]. Chitin is the common component of the cell wall of fungi. Dursun et al. have confirmed the ability of chitin to complex metal ions [73]. Volesky states that various polysaccharides, including cellulose, chitin, alginate, glycan, etc., existing in the cell walls of fungi, play a very important role in metal binding. Some functional groups having the ability to bind metal ions—in particular, carboxyl groups—have also been found. There is also evidence confirming that O–, N–, S–, or P– containing groups are directly involved in the bonding of some metals [5]. No works dealing with the tested wood-decaying fungus species have been published in the literature. However, other species, such as *Polypores versicolor* (bivalent ions of IIB group) [74], *Phanerochaete chrysosporium*, *Pleurotus ostreatus*, *Trametes versicolor* (bioaccumulation mechanism Cd, Pb and Zn) [75], and *Trametes versicolor polyporus* Cr (VI), have been tested [73].

Raw—the so-called virgin Merino wool—was used to study the biosorption of Cr (VI). Pure fiber consists of keratin, pigment, and chemically bound water. It is estimated that wool contains more than one hundred and seventy different proteins. The proteins present in wool consist of amino acids (i.e., they contain basic amino groups, –NH_2_, and acidic carboxyl groups –COOH). A few studies dealing with this issue have already been published in the professional literature. The authors were dealing with the ability to adsorb, for example, Hg (II), Cu (II) and Co (II) [76], and Cr (IV) [77]; the authors Dakiki et al. [78], in addition to other low-cost biosorbents, have also examined the possibility of using wool to remove Cr (VI). However, since inductively bound plasma spectrometry was used for hexavalent chromium analysis, instead of the commonly used spectrophotometric method with 1,5-diphenylcarbazide, the published results are likely to be biased. This significant shortcoming in the interpretation of data is also pointed out in the review of Miretzky et al. [79].

The aim of the study was to find the most suitable biosorbent possible, which would then be tested in a dynamic system, i.e., in continuous flow columns. The materials used as biosorbents were chosen to be readily available.

## 2. Materials and Methods

### 2.1. Biosorbent Preparation Methodology

A set of the following seven biosorbents was used to study the removal of hexavalent chromium from aqueous solution: *Fomitopsis pinicola*; a 1:1 mixture of Scots pine cones (*Pinus sylvestris*) and Norway spruce (*Picea abies*); peach stones (*Prunus persica*); apricot stones (*Prunus armeniaca*); walnut shells (*Juglans regia*); orange peels (*Citrus sinensis*); and raw, the so-called virgin Merino wool from young four-year-old sheep (Merino breed).

The pre-treatment was uniform for all biosorbents. In the first place, any coarse impurities were removed mechanically. Any eventual residues stuck to the sorption material were washed off with water and then rinsed with redistilled water. The clean samples were then pre-dried, in air, at room temperature (24 ± 2 °C), with occasional stirring, to prevent possible mould growth. In order to improve the binding interactions and also to improve the functionality and usability of the biosorbent in the biosorption process, the pre-dried biomass was also subjected to grain-size, heat, and chemical treatment.

Before the grain-size treatment, the material was first crushed (except for sheep wool), using a mobile crusher type Raptor 624, model HT 6523 (HECHT MOTORS s.r.o., Tehlovec, Czech Republic), and a laboratory mill type IKA A 11 basic was used for finer fractions, (IKA-Werke, Staufen im Breisgau, Germany). Before the sieving itself, the samples were dried at 105 ± 1 °C for the period of approximately 4 h (removal of the sticky effect). An ECOCELL standard dryer (BMT Medical Technology s.r.o., Brno, Czech Republic) was always used for the heat treatment of the biomass by drying. The grain treatment was performed by sieving to the required grain fractions (fr. 1.0/2.0 mm, fr. 0.5/1.0 mm, and fr. 0/0.5 mm), using a set of stainless steel sieves (Retsch GmbH, Haan, Germany).

**Figure 1:**
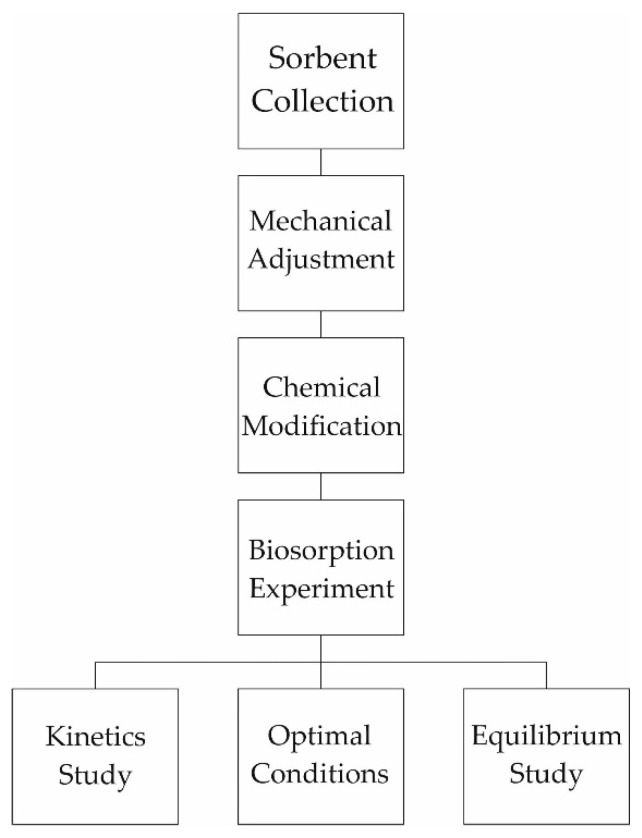
Scheme describing the biosorption experiment.

Hydrochloric acid with different molar concentrations, namely 0.1, 1.0, and 2.0 mol·L^−1^, was used for the chemical modification with respect to the nature of the studied metal. Then, 20 mL of the activating agent of the given concentration was added for each gram of the material. The biosorbent samples were activated by stirring in a shaking incubator type GFL 3031 (LAUDA DR. R. WOBSER GMBH & CO. KG, Burgwedel, Germany) at a constant speed of 150 rpm. The duration of activation adhered to the following time intervals for each grain size class and each molar concentration of activating agent: 15, 30, and 60 min. The supernatant was removed after chemical modification and activated material was repeatedly and systematically rinsed with redistilled water. The washing efficiency of the activating agent residues was checked and was carried out after washing, using a pH meter type ION 340i (Xylem Analytics Germany Sales GmbH & Co. KG, WTW, Weilhei, Germany). The modified biosorbents were dried at 105 ± 1 °C to constant weight. They were stored in plastic bags in a desiccator to ensure accurate weighing for further study of the biosorption process in a simple static batch mode. These chemically modified samples were used for further study. Scheme describing the biosorption experiment is in Figure 1.

### 2.2. Methodology of the Biosorption Modeling Process

To evaluate the biosorption and with regard to its practical application and the possibility of mutual comparison of the efficiency of the individual biosorbents, the achievement of the equilibrium state of biosorption was described by using uniform mathematical models: kinetic and equilibrium ones. All sorption studies were performed in static batch mode due to their simplicity. All experiments were performed in parallel three times on the same day. All the data, therefore, represent the mean of three independent experiments. The percentage error of the results was within 5–8%. The corrections to the possible adsorption of Cr (VI) on the inner surface of the container were made under similar conditions, namely the concentration, temperature, pH, speed of shaking, and time of sorption. The blind samples were processed under the same experimental conditions but in the absence of the adsorbent.

#### 2.2.1. Adsorption Kinetics Modeling

Adsorption kinetics modeling was used to determine the most ideal exposure time required for the biosorption of hexavalent chromium and to establish an equilibrium state between the two phases of biosorbent–sorbate. To design the sorption mechanism, it was important to first determine this time on the basis of an experimental study, using the following variables, i.e., activating agent concentration (HCl) and activation time, sorbate and sorbent concentration, particle size, stirring speed, temperature, and pH value. Each condition was studied separately to facilitate the possibility of comparing the results. The best results obtained from these experiments were then used to study the equilibrium model.

To model the biosorption kinetics, it was necessary to prepare a model solution of hexavalent chromium. The experiments took advantage of such an input concentration of Cr (VI) that was most suitable for the weighing of dry biosorbent (1.0000 ± 0.0200 g) and, at the same time, applicable to all biosorbents. This condition was met by the input concentration of 100 mg of hexavalent chromium mg·L^−1^. All experiments were performed by mixing 1.0000 ± 0.0200 g of biomass dried to constant weight with 0.05 L of Cr (VI) water solution at a concentration of 100 mg·L^−1^. The final concentration of biosorbent in the model solution of Cr (VI) was 20 mg·L^−1^. The experiments were performed in 0.1 L plastic sample cases (HDPE). The temperature during the experiment was constant at 23 ± 1 °C (controlled by a temperature sensor). The samples were shaken in a shaking incubator type GFL 3031 (LAUDA DR. R. WOBSER GMBH & CO. KG, Burgwedel, Germany) at 150 rpm. After a predetermined time interval (10, 20, 30, 40, 50, 60, 120, 180, and 240 min), the suspension was filtered through a PRAGOPOR 6 diaphragm filter (PRAGOCHEMA spol. Ltd., Prague, Czech Republic). The residual concentration of Cr (VI) in the filtrate was immediately determined. This procedure was applied to all types of modified biosorbents, i.e., different concentration of activating agent (0.1, 1.0, and 2.0 mg·L^−1^), different activation time (15, 30, and 60 min), and all studied grain fractions (fr. 1.0/2.0 mm, fr. 0.5/1.0 mm, and fr. 0/0.5 mm). This method was used to determine the ideal time to reach the equilibrium between the biosorbent and the sorbate and to find the highest sorption equilibrium capacity of the used sorbent.

The following relation (Equation (1)) was used to calculate the amount of Cr (VI) adsorbed at equilibrium per unit of adsorbent weight, i.e., the equilibrium sorption capacity of sorbent *q* [80,81]:

(1) $$q=\frac{V\left(c_{i}-c_{e}\right)}{S},$$

where we have the following:

*q*  adsorption capacity of the biosorbent, the amount of solute adsorbed at equilibrium per unit of adsorbent weight (mg·g^−1^),
*c_i_*  initial concentration of adsorbate in the solution (mg·L^−1^),
*c_e_*  equilibrium concentration of adsorbate in the solution (mg·L^−1^),
*S*  sorbent weight (g),
*V*  sorbate solution volume (L).

The percentage of the amount of removed Cr(VI) was expressed as removal efficiency (*R*%) and was calculated according to the Equation (2) [80,81]:

(2) $$R\left(\%\right)=\frac{c_{i}-c_{e}}{c_{i}}\times 100,$$

where the parameters of *c_i_* and *c_e_* have the same meaning as in the previous equation.

Two mathematical models were used to evaluate the experimental data: pseudo-first and pseudo-second-order. The assessment of the conformity between the experimental data and the calculated data applying the used mathematical model was performed on the basis of the evaluation of the obtained correlation coefficients (*R*^2^ values close to 1 or equal to 1). The relatively high value of *R*^2^ (*R*^2^ ≥ 0.950 was considered as a good one) indicated that the model successfully describes the adsorption kinetics of Cr (VI).

The following relation was used to calculate the speed parameters, using the pseudo-first-order kinetic Equation (3) [80,82]:

(3) $$\log\;\left(q-q_{t}\right)=\log q-\left(\frac{k_{1}}{2.303}\right)t,$$

where we have the following:

*q*  adsorption capacity, the amount of solute adsorbed at equilibrium per unit of adsorbent weight, (mg·g^−1^),
*q_t_*  amount of solute adsorbed at each time *t* (min) per unit of adsorbent weight, (mg·g^−1^),
*k*_1_ equilibrium speed constant of the first order pseudo-equation, (min^−1^),

and a pseudo-second-order kinetic Equation (4) [83]:

(4) $$\frac{t}{q_{t}}=\frac{1}{k_{2}q^{2}}+\frac{t}{q},$$

where *k*_2_ is the speed adsorption constant of the pseudo-second-order (g·mg^−1^·min^−1^), and the parameters of *q* and *qt* have the same meaning as in the previous relations.

The following relation was used to calculate the initial sorption speed, *h*, (mg·g^−1^·min^−1^) at time *t* → 0 (calculation was performed for *t* = 10 min) [5,84]:

(5) $$h=k_{2}q^{2},$$

Parameters have the same meaning as in the previous relations.

#### 2.2.2. Optimal Conditions

Optimal conditions were determined experimentally only for chemically modified biosorbents, which showed the highest efficiency of hexavalent chromium removal from the model solution in the shortest possible time when modeling the adsorption kinetics. It corresponded to the used exposure time, which depended on the biosorbent type.

The impact of the pH value on the biosorption process of hexavalent chromium was verified only for the pH range of 1.0–6.0, due to the nature of the studied metal and its dependence on the acidic environment. The individual pH values of the Cr (VI) model solutions were maintained by using buffers (pH of the used buffers 1.1, 2.0, 3.0, 4.0, 5.0, and 6.0), which were used to prepare the Cr (VI) model solution with a concentration of 100 mg·L^−1^. The other conditions always remained constant as in the case of kinetics modeling, as described above. The biosorbent concentration was 20 g·L^−1^, grain size fraction < 0.5 mm. The exposure time between the biosorbent and the adsorbate was always assessed in compliance with the best evaluated time within the scope of the kinetics study, which is why it was different according to the type of biosorbent.

The impact of the number of rotations on the Cr (VI) biosorption process was verified for the speed of 100, 150, 200, and 300 rotations per minute, in a shaking incubator type GFL 3031 (LAUDA DR. R. WOBSER GMBH & CO. KG, Burgwedel, Germany). Other conditions remained constant as in the case of kinetics modeling, as described above. The exposure time (contact time) between the biosorbent and the sorbate was in compliance with the best time evaluated within the scope of the kinetics study, which is why it also varied, depending on the type of biosorbent.

The impact of the concentration of the modified biosorbent on the biosorption process was studied for the following biosorbent weight of 1, 2, 3, and 4 g per 0.05 L of sorbate, i.e., the final concentration of biosorbent *c_s_* was 20, 40, 60, and 80 mg·L^−1^, in the presented order. The other conditions always remained constant as in the case of kinetics modeling. The exposure time between the biosorbent and the adsorbate was always in compliance with the best time evaluated within the scope of the kinetics study, which is why it varied according to the type of biosorbent.

The impact of temperature, as a physical parameter depending on the predominant action of the biosorption process, was tested for the temperature of 20, 30, and 40 ± 2 °C, depending on the exposure time. The experiments were performed in a shaking incubator type GFL 3031 (LAUDA DR. R. WOBSER GMBH & CO. KG, Burgwedel, Germany) and the temperature was controlled by a temperature sensor. The biosorbent was added to the Cr (VI) solution only when the temperature of the Cr (VI) solution had the desired value and, from this point on, the specific contact time (exposure time) was measured according to the used biosorbent. Other conditions remained constant as in the case of kinetics modeling.

The temperature dependence of the adsorption process is associated with several important thermodynamic parameters. The following thermodynamic equations were used to calculate the values of the thermodynamic parameters, such as the Gibbs energy change (Δ*G*^0^), the enthalpy change (Δ*H*^0^), and the entropy change (Δ*S*^0^) [85,86]:

(6) $$\Delta G^{0}=-RT\ln K$$

(7) $$\Delta H^{0}=\left[\frac{RT_{1}T_{2}}{\left(T_{2}-T_{1}\right)}\right]\ln\left(\frac{K_{2}}{K_{1}}\right)$$

(8) $$\Delta S^{0}=\left(\frac{\Delta H^{0}-\Delta G^{0}}{T}\right)$$

where we have the following:

**R**  universal gas constant (8.314 J·mol^−1^·K^−1^),
*T*  thermodynamic temperature (K),
*K*, *K*_1_, and *K*_2_ equilibrium constants at absolute temperatures of *T*, *T*_1_, and *T*_2_ (K).

The equilibrium constants, *K*, were calculated according to the following equation [86]:

(9) $$K=\left(\frac{q}{c_{e}}\right)\;\;$$

where we have the following:

*q*  adsorption capacity, amount of solute adsorbed at equilibrium per unit of adsorbent weight (mg·g^−1^),
*c_e_*  equilibrium concentration of adsorbate in solution (mg·L^−1^).

Based on the calculated thermodynamic parameters, it was determined whether it is an exothermic/endothermic process, whether it is chemisorption or rather physical adsorption, and whether the course of biosorption will take place spontaneously.

Hexavalent chromium adsorption experiments were performed to determine its equilibrium adsorption capacity under the most ideal set of conditions. They were found during the study of adsorption kinetics and optimal conditions for the process of biosorption of Cr (VI), using each studied set of biosorbents. The following constant conditions were kept for all the studied sorbents for the modeling of adsorption isotherms: pH = 1.1, grain size fraction fr. 0/0.5 mm, temperature 25 ± 2 °C, stirring speed 200 rpm, and chemically modified biosorbent concentration 20 g·L^−1^. The most suitable contact time (shaking time required to reach the equilibrium) used for isotherm modeling was determined from the kinetics study and varied depending on the type of used biosorbent.

A number of model Cr (VI) solutions with different input concentrations of Cr (VI) solutions were prepared (100, 200, 300, 400, 500, 600, 700, 900, and 1000 mg·L^−1^) to study the adsorption isotherms. The individual model solutions of the required concentration of Cr (VI) were prepared in a buffer with the value of pH = 1.1. The Langmuir and Freundlich models, both in linearized and non-linearized form, were used to describe the adsorption isotherms.

The Freundlich equation in nonlinear form is represented by the following relation for solution adsorption [87,88]:

(10) $$q=K_{F}c_{e}^{1/n}$$

where we have the following:

*K_F_*  Freundlich constant, also known as Freundlich capacity (mg·g^−1^),
1/*n*  Freundlich constant, indicates the intensity of adsorption,
*q*  amount of solute adsorbed per unit of adsorbent weight (mg·g^−1^),
*c_e_*  equilibrium concentration of the solute in the solution volume (mg·L^−1^).

Freundlich equation in linear form is expressed as follows [87,88]:

(11) $$\ln q=\ln K_{F}+\frac{1}{n}\ln c_{e}$$

The Freundlich constant *K_F_* is related to the maximum binding capacity, the constant *n* is used to describe the affinity of the binding sites to Cr (VI). Both Freundlich constants influence the adsorption isotherm model. The higher their values, the higher the adsorption capacity of the given metal. Both Freundlich constants were determined empirically.

Langmuir model in linear and nonlinear form is expressed by the following relations [89,90,91]:

(12) $$\frac{c_{e}}{q}=\frac{1}{Q_{max}K_{L}}+\frac{c_{e}}{Q_{max}}\;\mathrm{nonlinear}\;\mathrm{form}$$

(13) $$\frac{1}{q}=\frac{1}{Q_{max}}+\frac{1}{Q_{max}K_{L}}+\frac{1}{c_{e}}\;\mathrm{linear}\;\mathrm{form}$$

where we have the following:

*q*  adsorption capacity, amount of solute adsorbed per unit of adsorbent weight (mg·g^−1^),
*Q_max_*  maximum metal adsorption under constant conditions (mg·g^−1^);
*K_L_*  Langmuir constant related to metal–sorbent affinity;
*c_e_*  equilibrium concentration of adsorbate in solution (mg·L^−1^).

The Langmuir constant *K_L_* is determined empirically and expresses the affinity of the binding sites to Cr (VI).

The following relation was used to calculate the separation factor *R_L_* [92]:

(14) $$R_{L}=\frac{1}{1+bc_{i}}$$

where we have the following:

*b*  parameter from the straight-line equation,
*c_i_*  initial metal concentration in solution (mg·L^−1^).

### 2.3. Cr (VI) Analysis Methodology

All working solutions with different concentrations of hexavalent chromium were obtained by sequential dilution of the stock solution with the concentration of 1 g·L^−1^, which was prepared by dissolving dipotassium dichromate of analytical purity (K_2_Cr_2_O_7_ p.a., Penta Ltd., Prague, Czech Republic) in redistilled water. A newly prepared solution was always used for each experiment. A buffer solution with the appropriate pH value (1.1, 2.0, 3.0, 4.0, 5.0, and 6.0) was used as the solvent to prepare the solutions, which were supposed to have a specific pH value. This ensured a constant value in the solution. The pH value was verified, using a table pH meter type ION 340i (Xylem Analytics Germany Sales GmbH & Co. KG, WTW, Weilhei, Germany).

The input concentration of hexavalent chromium *c_i_* and the equilibrium residual concentration of hexavalent chromium in the filtrate after biosorption were analyzed, using the spectrophotometric method by producing a violet color with 1,5-diphenylcarbazide on a DR 2800 spectrophotometer (Hach Lange GmbH, Düsseldorf, Germany). The principle of determining the content of hexavalent chromium in aqueous solution is based on the chemical reaction of 1,5-diphenylcarbazide to 1,5-diphenylcarbazone, which forms a red-violet-colored complex with chromates or dichromates in an acidic aqueous environment. The absorbance of the colored solution is in linear relation with the concentration of hexavalent chromium and is measured photometrically at a wavelength of 540 nm [93]. The total amount of chromium present in the solution was determined by the same procedure described for the determination of Cr (VI) after the oxidation of the present Cr (III) with an excess of potassium permanganate (KMnO_4_ p.a., Penta Ltd., Prague, Czech Republic). The adsorption capacity claimed in the results was calculated from the difference between the initial concentration of hexavalent chromium ci and the final concentration of total chromium c. The difference between the final concentration of total chromium and the final concentration of Cr (VI) represents the concentration of Cr (III).

## 3. Results and Discussion

To evaluate the efficiency of biosorption, it is important to consider two crucial physical–chemical aspects of the process, kinetics, and equilibrium. Since the aim of the whole study was to select the best biosorbent/biosorbents that would be useful for removing Cr (VI) from aqueous solution, one of the prerequisites was to keep the conditions of the individual experiments as uniform as possible. This is the only way how to objectively compare the biosorbents and select the best ones, which can be further studied in a dynamic system, i.e., in continuous flow columns. The static batch biosorption tests that were studied provide only basic information concerning, especially, the efficiency of chromium biosorption, using the given biosorbent. However, the continuous mode of operation is preferred in most industrial wastewater treatment plants.

Untreated biosorbents have a much lower adsorption capacity than chemically modified ones. That is why their pre-treatment was used first in order to increase the Cr (VI) removal efficiency by means of biosorbents. Chemical activation was chosen for the sample pre-treatment in case of the experiments presented in this article. The used plant biomaterials, the main structural components of which are lignin, cellulose, and other organic macromolecules, thus primarily contain weak acidic and basic groups on their surface. With regard to the speciation of hexavalent chromium, hydrochloric acid of various concentrations (0.1, 1.0, and 2.0 mol·L^−1^) was used for 15, 30, and 60 min, to increase the positive charge on the surface of the biosorbents, and thus to increase the available sites for the adsorption of chromium anions. Chemical modification using HCl acid with the concentration of 0.1 mol·L^−1^ was not very effective, which is why the results are not presented or discussed here. As for the length of the activation time, it was individual and varied according to the type of biosorbent. No correlation was found between the concentration of the applied activating agent, the activation time, and the type of biosorbent. In summary, the individual results obtained during the testing are shown in Table 1, where the information presented for each biosorbent with a grain size fraction of 0/0.5 mm consists of the concentration of the used activating agent, the activation time, and the amount of sorbed Cr (VI) per 1.0000 g of biosorbents; *q_t_*, including the exposure time, *t*, between the biosorbent and the sorbate at which the respective highest sorption capacity, *q_t_*, was found. *Fomitopsis pinicola* proved to be the best in removing all Cr (VI) from the solution within one hour, and its efficiency was therefore 100%, without adjusting the pH value of its model solution. Good results were also obtained in the case of the mixture of cones, where the maximum sorption (100%) was already reached after 40 min.

Mass transfer is determined depending on the particle size and may, to some extent, affect the efficiency of biosorption. Three groups of grain fractions were studied (fr. 1.0/2.0, fr. 0.5/1.0, and fr. 0/0.5 mm). The entire share of fraction 0/0.5 mm was used in order to facilitate the sorting of the grain sizes and also because the particles of smaller dimensions could be poorly wetted by the sorbate (slower sedimentation properties). With respect to the used biosorbents, it was also presumed that sorption could be controlled by ion exchange as well, which does not depend on the accessible surface. Based on the best sorption efficiency, the particle size of 0–0.5 mm was chosen as the limiting parameter for all biosorbents. This is mainly due to the larger active surface area of the small particles, which means that more binding sites are made available. That is why the particles larger than 2.0 mm were not studied because, when compared to smaller particles, they contribute to increasing the diffusion resistance to mass transfer more. It must be noted that this parameter is not studied systematically and each author uses his own right conditions. This leads to inconsistencies (as a result of the proven effect of particle size on the biosorption mechanism) in cases when the authors compare their results with the findings of other colleagues who work with different parameters. Another disadvantage is that the results are reported as percentage efficiency and not by means of the equilibrium sorption capacity *q* in mg·g^−1^. With regard to this fact, the results obtained in this work are not compared with similar works by other authors due to the lack of objectivity.

In the biosorption process, the pH value is one of the most important parameters that can significantly affect the entire biosorption process. The pH value of aqueous solution affects the speciation of chromium and also the dissociation of active functional groups (–OH, –COOH, and –NH_2_) present on the surface of the biosorbent. For this reason, the adsorption of chromium is critically linked to the pH value of the solution. At low pH values, the functional groups on the surface of the biomaterial are protonated, thus limiting the access of cationic types due to detachment forces. On the other hand, increasing pH value is accompanied by decreasing level of protonation and the functional groups therefore become negatively charged (pH > pKA) [94].

The aqueous solution of Cr (VI) exists in five anionic forms: H_2_CrO_4_, HCrO_4_^−^, CrO_4_^2−^, HCr_2_O_7_^−^, Cr_2_O_7_^2−^, the distribution of which depends not only on the pH value but also on the total chromium concentration. At pH values from 2.0 to 6.0, Cr (VI) ions in the solution are likely to be in the form of HCrO_4_^−^ and Cr_2_O_7_^2−^. At lower pH values (pH < 2.0), the main types are Cr_4_O_13_^2−^ and Cr_3_O_10_^2−^. These anionic types can then be sorbed to protonated active sites that are present on the surface of the biosorbent [95].

With respect to the speciation of the studied metal and the fact that the sorption of metals in the form of anions is most effective in an acidic environment, only the pH range from 1.1 to 6.0 was verified to study the impact of pH. The pH values > 6.0 were not studied here, because there is no confirmation of Cr (VI) adsorption at pH values higher than 6.0, due to competition of HCrO^4−^, Cr_2_O_7_^2−^, and OH^−^ anions for the adsorption sites. The consumption of H^+^ protons through Cr (VI) reduction leads to an increase in the pH value of the solution. Its control in the system during the biosorption is therefore very important. This was prevented by using the buffers, which ensured that the pH value was constant throughout the experiment.

It was experimentally ascertained in the study of the impact of the pH value of the used model solution on the removal of hexavalent chromium depending on the exposure time that lowering the pH of sorbate to 1.1–2.0 significantly improved the sorption capacity of all the tested biomass. In some cases, the exposure time between the biosorbent and the sorbate was even shortened, which is beneficial especially for its further potential application in practice. The contact time was most significantly shortened in case of orange peels (by 80 min), *Fomitopsis pinicola* (by 50 min), and a mixture of cones (by 30 min). As far as the remaining biosorbents are concerned, only the biosorption efficiency has improved (see Table 2). The values of the sorption capacity qt are presented for the exposure time. The highest *qt* values were found during the adjustment of the pH of the model solution to the value of 1.1, in order to make an objective assessment of the impact of the pH value possible.

Based on the results obtained during the study of the impact of pH on Cr (VI) removal, it was also confirmed that the removal rate was significantly dependent on the pH and decreased with the increasing pH value. The mechanism of hexavalent chromium biosorption in an acidic environment was probably its reduction to trivalent chromium through direct reduction reactions and adsorption of trivalent chromium ions at higher pH values. At a very low pH value (pH = 1.1), hexavalent chromium anions reduced to trivalent chromium ions, but they will be poorly adsorbed by the biosorbent due to electrostatic repulsive forces. hexavalent chromium is reduced to trivalent chromium in the aqueous phase (direct mechanism) upon contact of hexavalent chromium with the electron-donor group of biomaterials, which has a lower value of the reduction potential than hexavalent chromium. The trivalent chromium ions then remain in aqueous solution or form complexes with Cr-bonding groups which are found on the surface of the biosorbent [4,96]. Since no measurable Cr (III) content was found in the filtrate (pH < 3.0) during the experiments, it can be concluded that there is a possibility of complexation contribution in the removal of hexavalent chromium.

The vast majority of authors also state that the highest efficiency in the removal of hexavalent chromium was achieved within the range of pH = 1.0–2.0 [45]. On the other hand, it should be noted that many studies also state that hexavalent chromium was removed from aqueous solutions by biomaterials through adsorption between positively charged adsorption sites on the surface of the adsorbent and anionic Cr (VI). Mohan et al. [97] point out that the conclusions concerning the removal of Cr (VI), which are allegedly carried out by an electrostatic mechanism, could arise due to a misinterpretation of the acquired information. This is caused by errors in the measurement of chromium speciation in the aqueous phase, insufficient contact time required to reach the equilibrium and also the lack of information on the oxidation state of chromium bound to biomaterials. At present, adsorption coupled with reduction is generally considered to be the real mechanism of adsorption of Cr (VI), using biomass under acidic conditions [4,82,84]. Park et al. state that Cr (VI) can be removed from aqueous solution by natural biomaterial both by means of direct and indirect reduction mechanisms [81]. The indirect mechanism of reduction is rather more complicated and basically consists of three steps. First, the anionic Cr (VI) binds to positively charged groups on the surface of biomaterials (amino groups and carboxyl groups). In the second step, it is reduced to Cr (III) by means of adjacent electron-donor groups, which is then accompanied by the release of Cr (III) into the aqueous phase due to detachment forces between the positively charged Cr (III) and positively charged groups on the surface of the biomaterials or complexes of Cr (III) with adjacent groups.

The kinetic biosorption study described the speed of removal of hexavalent chromium, which is controlled by the contact time between the biosorbent and the sorbate. Two kinetic models were used to analyze the experimental data: pseudo-first and pseudo-second-order. These models were used to clarify the mechanism of adsorption and its possible speed control steps, which include mass transport and chemical reactions. The speed constants calculated by means of the used models were evaluated depending on the exposure time at ambient temperature and the most suitable concentration (100 mg·L^−1^). Since there is a significant impact of pH on the whole biosorption process, the kinetic parameters were also compared both for the experiments performed in an acidic environment (pH = 1.1) and solutions without pH adjustment.

The total time required to reach the adsorption equilibrium of the Cr (VI) concentration ranges from minutes to hours. In general, the solid–liquid adsorption process could be described by the following three steps:

1. External diffusion-transport of adsorbate from the solution by means of a liquid film to the outer surface of the sorbent.
2. Internal diffusion-transport of adsorbate from the outer surface of the adsorbent to the pores of the sorbent.
3. The adsorbate is adsorbed to the active groups on the inner and outer surface of the adsorbent.

By applying the pseudo-first-order model, it was revealed that the values of correlation coefficients don’t meet the condition (R^2^ > 0.950) and the model in question is not suitable for the description of the experimental data. In addition, the calculated theoretical values of the sorption capacity *q_cal_* obtained by this method contrasted with the experimental values of *q_exp_*. Based on this finding, the reactions could not be classified as first-order reactions for any of the tested biosorbents. One explanation, with respect to the differences between the experimental and theoretical values of *q*, may be the fact that there is a time lag during the sorption process, probably due to the thin layer or the effect of external resistance at the beginning of biosorption. Since this model, which is suitable for homogeneous surfaces, was not applicable to the studied biosorbents, it can be concluded that there will probably be more sorption sites and other mass transfer effects.

The pseudo-second-order model is based on the assumption that the speed-limiting step can be represented by chemical sorption involving valence forces through electron sharing or exchange between the adsorbent and the adsorbate [32,98,99]. The equilibrium adsorption capacities, *q*, that were obtained by using this model during the performed experiments were also much more reasonable than those obtained by using the pseudo-first-order model. When comparing the expected *q_cal_* results with the experimental data, there was a very good conformity (see Table 3). According to the pseudo-second-order model, the speed constant *k*_2_ is related to the overall rate of the sorption process. The data obtained confirm that the speed at pH = 1.1 is much higher than those obtained during the experiments where the pH value had not been adjusted. Even in the case of *Fomitopsis pinicola* and a mixture of cones, there was a multiple increase in the total reaction speed. More importantly, the initial speed of Cr (VI) removal also increased sharply in an acidic environment (pH = 1.1). Based on the used pseudo-second-order model, it was found that 6 g of Cr (VI) per gram of sorbent were absorbed per minute, using *Fomitopsis pinicola* under the given conditions during the first ten minutes. In the case of a mixture of cones, it was 1000 mg. This is a very important factor when designing and optimizing the processes in the industry.

On the contrary, apricot stones and peach stones seem to be the least suitable, based on the kinetic parameters obtained within the scope of the pseudo-second-order model. The speed at which Cr (VI) will be removed from the diluted aqueous solution by the biosorbents represents an important factor for the application when taking into account the water quality control. The capacity of the sorbent to receive sorbate, i.e., the state in which the equilibrium is reached, also determines its lifetime to great extent. It is therefore important to determine the speed at which contaminants are removed from the aqueous solution on the basis of the experiments, in order to be able to design the sorption process [100]. The results of the experiments support the theory of such a sorption model, during which the speed limiting factor for hexavalent chromium biosorption is chemisorption, which involves valence forces through the sharing or exchange of electrons between the adsorbent and the metal ions [101].

The speed of mass transfer from the Cr (VI) solution to the solid surface of the biosorbent also to some extent depends on the stirring intensity. The stirring speed, therefore, plays a role in the transfer of the chromium mass from the solution to the biosorbent surface as well. The experiments involved the test of rotation speed within the range of 100–300 rpm while maintaining the other parameters of biosorption constant (*c_i_* = 100 mg·L^−1^; *t* = 25 ± 2 °C; *c_s_* = 20 g·L^−1^). However, no significant effect of the stirring speed was found in the given speed range. The rate of 200 rpm has been chosen to study the equilibrium of biosorption because it provided the best homogeneity of the mixture suspension. Higher rates of rotation have been ruled out due to the possible formation of vortexes during high mixing rates. There was a risk that the suspension might not be homogeneous and that the adsorption of Cr (VI) could be adversely affected as a result of that.

The impact of biosorbent concentration on the removal of Cr (VI) from aqueous solution, while maintaining the other biosorption parameters constant (*c_i_* = 100 mg·L^−1^; *t* = 25 ± 2 °C; stirring rate 150 rpm, without pH adjustment), was studied as well. The experiments were performed for the concentration ranges of the biosorbent from 20 to 80 g·L^−1^, depending on the exposure time. Cr (VI) removal decreased with increasing biosorbent dose. Higher adsorption in the case of a lower concentration of biosorbent may occur as a result of an increased ratio of Cr (VI) to biosorbent, which, however, decreases due to an increase of the dry biomass dose. It was also revealed that the percentage of Cr (VI) removal increased with increasing biosorbent dose, but only above a certain value when the percentage of removal had already reached the saturation level. This phenomenon probably occurs due to the resistance to mass transfer of Cr (VI) from the solution at a high dose of biosorbent. Taking into account the fact that the study of the impact of biosorbent concentration on the course of biosorption in the case of orange peel revealed certain problems (formation of dense gel mass) when its concentration was increasing, the concentration of 20 g·L^−1^ was chosen as the limiting parameter for further study of all tested biomass.

Influencing the biosorption process by temperature depends mainly on the predominant process, which represents the essence of the biosorbent–metal bond forces. Thermodynamic considerations related to the adsorption process must therefore be made in order to decide whether the process is spontaneous or not.

The thermodynamic study also included the study of the effect of temperature on the removal of Cr (VI) within the range of temperatures from 293 to 313 K (i.e., 20–40 °C). Negative values of the change of Gibbs free energy Δ*G*^0^ at all temperatures indicate the feasibility and spontaneity of the adsorption process. Spontaneous processes usually also correspond to an increase in the positive value of the change in entropy Δ*S*^0^, which was confirmed as well. There is no significant decrease in the change of Gibbs energy with increasing temperature. This indicates that the higher temperature will not have a significant effect on the course of biosorption. In general, the Gibbs energy change for multilayer adsorption should be higher than −20 kJ·mol^−1^ and lower than zero [102]. Since the change of Gibbs energy in all studied biosorbents was lower than −20 kJ·mol^−1^, it will probably not be multilayer adsorption.

A negative value of Δ*H°* also indicates that the adsorption will in most cases have a slightly exothermic character within the studied temperature range. The highest value of enthalpy was found in case of walnut shells (Δ*H°* = −992 kJ·mol^−1^) and *Fomitopsis pinicola* (Δ*H°* = −179 kJ·mol^−1^). This means that the adsorption capacity of the biosorbents will more likely increase with decreasing temperature. Similar conclusions were reached, for example, by Kapoor et al. [103]. On the contrary, the lowest value of the enthalpy change (Δ*H°* = −61 kJ·mol^−1^) was found in case of peach stones and a mixture of cones (Δ*H°* = −62 kJ·mol^−1^). No significant differences in Δ*G*^0^ values depending on temperature were found. The sorption capacity increase did not exceed 5–10%. This could indicate that, under the given conditions, the temperature will not have a significant effect on the course of removal of Cr (VI) from the aqueous solution.

In general, in the case of chemisorption, increasing the temperature will positively affect the adsorption of the metal from the solution, while in the case of physical adsorption, the amount of eliminated metal will decrease with increasing temperature. However, temperature often does not affect the biosorption process in any way. Within the scope of the study of other factors, the effect of temperature on the overall biosorption process is also generally considered to be insignificant.

Since the study of kinetics had revealed that the experimental data could be well described by a pseudo-second-order kinetic equation, it was concluded that chemisorption can be the limiting step of biosorption. Nevertheless, based on the study of thermodynamics, it is necessary to supplement this conclusion with the assumption that it will probably not be the only mechanism that will take place during the biosorption. If this were the case, the rising temperature would have to positively affect the adsorption of metal from the solution. There was no decrease in the sorption capacity with increasing temperature, which means it cannot be physical adsorption either. Several mechanisms are likely to be involved in the elimination of Cr (VI), which is why the nature of the predominant control mechanism of biosorption cannot be strictly determined. It is also impossible to neglect a large number of functional groups in chitin, cellulose, and other macromolecules present in the biosorbents. They can be very effective for the complexation of chromium ions [104] and also play a significant role in the mechanism of biosorption.

A positive value of Δ*S*^0^ indicates the fact that during the sorption the randomness at the solid–liquid interface increases during the adsorption process, which can mean that Cr (VI) ions in the solution replace certain water molecules previously adsorbed on the adsorbent surface. The calculated values of the thermodynamic parameters are presented in Table 4.

Because the sorption processes tend to be exothermic, and because the sorption performance can vary depending on temperature, constant temperature is the basic requirement during biosorption. The temperature of 25 ± 2 °C was chosen for further study as the limiting factor based on the evaluation of the study of the impact of temperature on the course of biosorption and with regard to further use in industrial practice. Many other authors also use temperature within the range of 20–25 °C, in the study of metal biosorption, using biomaterials [105,106]. This means that temperature may be the parameter that will affect the sorption of metal ions; however, most published studies have concluded that the effect of temperature is only limited, and only within a certain range of temperatures. This fact leads to the conclusion that one of the mechanisms responsible for the sorption process may also be the ion exchange [53].

The comparison of the sorption capacity of the sorption of one metal can be executed best on the basis of the sorption isotherm for one metal. In order to make the comparison of two or more sorbents objective, the sorption was always performed under the same conditions. These were limited by the environmental factors under which the biosorption could take place (pH value and temperature). The adsorption data were described by using two models: Langmuir and Freundlich adsorption isotherms. Both models have been widely used by other authors to examine the sorption equilibrium between a metal solution and a solid biomass phase [41,49,107,108].

Although the Langmuir model does not provide any explanation for the mechanistic aspects of biosorption, it can provide information on the uptake capacity and is able to reflect the usual behavior of the equilibrium sorption process. Langmuir assumed that the forces exerted by chemically unsaturated surface atoms (total number of binding sites) do not extend beyond the diameter of one sorbed molecule, and the sorption is therefore limited to one layer only. The Langmuir model is the most commonly used one in practice, because it contains two useful and easily imaginable parameters (*Q_max_* and K_L_), which are easy to understand because they reflect two important characteristics of the sorption system. However, when applying it to molecular types (biosorbents), it is necessary to keep in mind that the accepted assumptions of these original relationships actually come from the experiments performed with activated carbon, as a solid sorbent. The monomolecular layer on which the sorbates are deposited relies on sorption on the surface, which is not always true in case of biosorption [5,109,110,111]. Experience from batch studies has shown that Cr (VI) removal decreased with increasing initial Cr (VI) concentration, suggesting that the adsorbents had a limited number of sites that were already saturated above certain concentration, and there was no further adsorption. The calculated parameters of both models for the maximum concentration (1000 mg·L^−1^) are given in Table 5.

The value of the maximum adsorption capacity, *Q_max_*, can also be interpreted as the total number of binding sites available for biosorption and the adsorption capacity at the best exposure time, *q_t_*, as the number of binding sites that are occupied by sorbate at a given input concentration, *c*_0_. The highest value of the maximum sorption capacity (*Q_max_* = 45 mg·g^−1^) was calculated for *Fomitopsis pinicola*, using the Langmuir model. On the other hand, the lowest value (*Q_max_* = 26 mg·g^−1^) was calculated for peach stones. The experimental data obtained within the scope of the biosorption equilibrium study were well described using the linear Langmuir model and were in a relatively good conformity with the calculated values.

The initial gradient is another important characteristic of the sorption isotherm curve. The steep initial gradient of the sorption isotherm indicates biosorbent that will have a good sorption capacity for the sorbate within the low residual concentration range *c_e_*. This means that the biosorbent will have a high affinity for the sorbed type, i.e., Cr (VI). This affinity is indicated by the Langmuir constant, *K_L_*. The lower *K_L_* value, the higher the sorbent affinity should be for the sorbate. In general, for “good” sorbents, it is necessary to find a high value of *Q_max_* and a steep gradient of the initial sorption isotherm, i.e., low values of the Langmuir parameter, *K_L_* [111,112]. However, none of the studied biosorbents met this condition. Although the highest initial gradient of the sorption isotherm was calculated for peach and apricot stones, the values of the maximum sorption capacity were the lowest for the biosorbents in question.

In the case of peach stones, the experimental data can also be described by using the Freundlich model, which means that, apart from chemisorptions, the biosorption will probably also involve physical sorption. However, if we evaluate the entire area of the graph, which is always important, it can be concluded that for the concentrations of Cr (VI) lower than 100 mg·L^−1^ *Fomitopsis pinicola* will show the highest sorption efficiency of all the studied biosorbents. This is very important, for example, in case the biosorbent is to be applied at low residual concentrations of sorbate, e.g., if it is necessary to comply with the permissible value for the discharge of waste chromate water. Shells of Persian walnut and fleece will be suitable for pollution concentration ranges of up to 200 mg·L^−1^. Although apricot and peach stones do not have such a good sorption capacity for lower sorbate concentrations, they have the ability to sorb Cr (VI) in a wide concentration range, i.e., from 100 to almost 800 mg·L^−1^ (in the case of apricot stones).

The separation factor *R_L_* is another parameter that can be calculated from the Langmuir model. If the *R_L_* value is a positive number, its magnitude determines the feasibility of the biosorption process. If *R_L_* > 1, the adsorption will be unfavorable; *R_L_* = 1 linear; 0 < *R_L_* < 1 favorable; and *R_L_* = 0 the adsorption will be irreversible. Based on this parameter calculated for the initial concentration of 1000 mg·L^−1^, it can be stated that the biosorption of these biosorbents will be irreversible, since the values of the separation coefficient were zero for orange peels, apricot, and peach stones, which is not favorable. This is because it is desirable for the process to be reversible in order to recover the sorbate as part of the biosorbent recovery. This is why these sorbents were not studied any further. The course of biosorption, using a mixture of cones, should be linear within the studied concentration range. Although the remaining biosorbents meet the condition for a favorable biosorption course 0 < *R_L_* < 1, the values are relatively low. This means that they will be more suitable for lower concentrations of Cr (VI). This is the reason why they will be further studied within the dynamic system (continuous flow columns). The adsorption data for chemically modified sheep fleece and peach stones can also be easily described by using the Freundlich model. Based on the value of the Freundlich constant (*n* = 2.58 and *n* = 2.56), which is much higher than 0 and is within the range of 2–10, it can be assumed that biosorption, using these biosorbents, could be good. The physical sorption will probably contribute to the biosorption process as well.

Conventional methods for removing metal ions, such as chemical coagulation or membrane filtration, are extremely expensive when treating large amounts of contaminated water, and they are often inefficient, especially for low metal concentrations (incomplete metal removal). There are also large amounts of sludge and other toxic products, which require subsequent disposal. Here, we see the benefit of the application of low-cost biosorbents that are very suitable for the final treatment of already treated wastewater. There is also a possibility of the application and other alternative pre-treatments of selected biosorbents, such as their carbonization, which could positively affect the mechanism of biosorption. Some authors already have very good experience with this pre-treatment [10,30,33,34,67,71,91,92,97,110]. Continuous mode of operation is preferred in most industrial wastewater treatment plants, and that is why the selected biosorbents will be further studied in a continuous flow mode.

## 4. Conclusions

This article presents the results and findings of a long-term experimental research focused on the possibility of removing hexavalent chromium from the aqueous environment, using low-cost waste biomaterial. In the first phase, a set of seven biosorbents was used for the study: (1) *Fomitopsis pinicola*, (2) a mixture of Scots pine cones (*Pinus sylvestris*) and Norway spruce (*Picea abies*), (3) peach stones (*Prunus persica*), (4) apricot stones (*Prunus armeniaca*), (5) walnut shells (*Juglans regia*), (6) orange peel (*Citrus sinensis*), and (7) Merino sheep wool, which were studied in simple static batch systems.

The adsorption data in the study of biosorption equilibrium were described by using two models: Langmuir and Freundlich adsorption isotherms.

Based on the Langmuir model, which was practically applicable to describe the adsorption data of all biosorbents, it was found that, for concentrations of Cr (VI) that are lower than 100 mg·L^−1^, *Fomitopsis pinicola* will show the highest sorption efficiency of all the studied biosorbents. This is important in the case where the biosorbent is to be applied at low residual sorbate concentrations, e.g., it would be necessary to comply with the permissible value for the discharge of waste chromate water.

For the contamination concentration range of up to 200 mg·L^−1^, the shells of walnut and sheep fleece will be suitable. Although apricot and peach stones do not have such a good sorption capacity for lower sorbate concentrations, they have the ability to sorb Cr (VI) in a wide concentration range, i.e., from 100 to almost 800 mg·L^−1^ (in the case of apricot stones).

## Figures and Tables

**Table 1:** Results of chemical modification of low-cost materials, using HCl acid for different grain fractions, activating agent concentration, and activation time, including the contact time.

Sorbent	HCl Concentration mol·L^−1^	Activation Time min	Grain Size mm	*q_t_* mg·g^−1^	Exposure Time (*t*) min
Orange peels	1.0	30	<0.5	3.81	120
*Fomitopsis pinicola*	1.0	60	<0.5	**5.07**	60
Mixture of cones	2.0	60	<0.5	**4.70**	40
Peach stones	1.0	30	<0.5	1.83	180
Apricot stones	2.0	15	<0.5	0.88	180
Walnut shells	1.0	30	<0.5	2.74	180
Fleece	2.0	15	x	**4.44**	180

Standard conditions: *c_s_* = 20 g·L^−1^; *c_i_* = 100 mg·L^−1^; without pH modification; stirring speed 150 rpm; *t* = 25 ± 2 °C; sorbents that were best for removing Cr (VI) from aqueous solution are indicated in bold.

**Table 2:** Impact of pH value on the exposure time and efficiency of Cr (VI) removal from aqueous solution.

Biosorbent	The Adsorption Capacity *q_t_* mg·g^−1^ pH = 1.1	The Adsorption Capacity *q_t_* mg·g^−1^ without pH Modification
Orange peels	*q*_40_ = 5.0	*q*_40_ = 1.7
*Fomitopsis pinicola pinicola*	*q*_10_ = 5.0	*q*_10_ = 2.4
Mixture of cones	*q*_10_ = 5.1	*q*_10_ = 3.5
Peach stones	*q*_180_ = 4.6	*q*_180_ = 1.8
Apricot stones	*q*_180_ = 3.7	*q*_180_ = 0.9
Walnut shells	*q*_60_ = 5.0	*q*_60_ = 2.0
Fleece	*q*_180_ = 4.7	*q*_180_ = 1.2

Standard conditions: *c_s_* = 20 g·L^−1^; *c_i_* = 100 mg·L^−1^; stirring speed 150 rpm; *t* = 25 ± 2 °C.

**Table 3:** Kinetic parameters calculated from the pseudo-second-order model for the studied biosorbents (*c_i_* = 100 mg·L^−1^; *t* = 25 ± 2 °C; stirring speed 150 rpm; *c_s_* = 20 g·L^−1^).

Biosorbent	Without pH Modification					pH = 1.1				
	*q_exp_*	*q_theor_*	*k*_2_	*h*_10_	R^2^	*q_exp_*	*q_theor_*	*k*_2_	*h*_10_	R^2^
Orange peel	3.8	2.4	0.18	0.3	0.722	5.0	5.0	7.13	168	**0.999**
*Fomitopsis pinicola*	5.0	5.5	0.53	3.1	**0.995**	5.0	5.0	263	6190	**1.000**
Mixture of cones	4.7	5.3	0.68	3.7	**1.000**	5.1	5.1	55.2	1321	**1.000**
Peach stones	1.8	1.8	0.10	0.1	**0.978**	4.6	5.0	0.36	1.38	**0.995**
Apricot stones	0.9	1.1	0.02	0.0	**0.968**	3.7	3.8	0.38	1.18	**0.999**
Walnut shells	2.7	3.0	0.12	0.2	**0.987**	5.0	5.2	0.57	3.70	**0.993**
Fleece	4.4	4.8	0.20	0.3	**0.993**	4.7	4.8	0.74	10.86	**0.999**

Notes: units of the presented parameters are *q* = mg·g^−1^, *k*_2_ = g·mg^−1^·min^−1^, and *h*_10_ = mg·g^−1^·min^−1^; the values of the correlation coefficients R^2^ ≥ 0.950 are marked in bold.

**Table 4:** Values of thermodynamic parameters Δ*G*^0^, Δ*H*^0^, and Δ*S*^0^, describing the removal of Cr (VI), using the tested chemically modified biosorbents.

Biosorbent	*T* K	Δ*G*^0^ kJ·mol^−1^	Δ*H*^0^ kJ·mol^−1^	Δ*S*^0^ J·mol^−1^·K^−1^	R^2^
Orange peel	293	−5.46	−74	18.6	0.902
	303	−3.57		11.8	**1.000**
	313	−7.53		24.1	0.897
*Fomitopsis pinicola*	293	−4.18	−179	14.3	**1.000**
	303	−6.33		20.9	**0.998**
	313	−9.00		28.8	**1.000**
Mixture of cones	293	−4.45	−62	15.2	**0.995**
	303	−5.43		17.9	**0.975**
	313	−9.47		30.3	0.926
Peach stones	293	−5.57	−61	19.0	0.949
	303	−5.63		18.6	0.933
	313	−12.35		39.4	**0.998**
Apricot stones	293	−2.37	−80	8.1	0.903
	303	−15.69		51.7	**0.998**
	313	−14.28		45.6	0.883
Walnut shells	293	−6.63	−992	22.6	**0.952**
	303	−10.76		35.5	**1.000**
	313	−9.54		30.5	0.923
Fleece	293	−5.19	−71	17.7	0.916
	303	−6.32		20.9	**0.961**
	313	−12.74		40.7	**0.984**

Standard conditions: *c_i_* = 100 mg·L^−1^; stirring speed 150 rpm; *c_s_* = 20 g·L^−1^; without pH adjustment; exposure time 10, 20, and 30 min. The values of the correlation coefficients *R*^2^ ≥ 0.950 are marked in bold.

**Table 5:** Constants of isothermal models and correlation coefficients of Cr (VI) adsorption, using the studied chemically modified biosorbents.

	Langmuir Model					Freundlich Model		
	*q_t_* mg·g^−1^	*Q_max_* Mg g^−1^	*K_L_* L mg^−1^	*R_L_* c_i_ = 1000 mg·L^−1^	*R* ^2^	*K_F_*	*n*	*R* ^2^
Orange peel	31.3	31.4	0.055	0.00	**0.988**	3.44	14.51	0.841
*Fomitopsis pinicola*	46.2	45.1	1.116	0.05	**0.993**	8.13	2.06	0.842
Mixture of cones	41.4	41.0	0.453	1.00	**0.996**	10.03	3.06	0.763
Peach stones	23.2	25.5	0.017	0.00	**0.994**	2.31	2.58	**0.963**
Apricot stones	10.0	10.4	0.020	0.00	**0.992**	1.97	3.95	0.829
Walnut shells	37.5	37.7	0.141	0.06	**0.998**	8.05	3.06	0.844
Fleece	36.5	40.3	0.036	0.01	**0.994**	3.86	2.26	**0.977**

Notes: *c_s_* = 20 g·L^−1^; pH = 1.1; stirring speed 200 rpm, *t* = 25 ± 2 °C; the best exposure time for each sorbent and maximum concentration 1000 mg·L^−1^. Correlation coefficient values *R*^2^ ≥ 0.950 are indicated in bold.

## Data Availability

Data is contained within the article or supplementary material.

## Supplementary Materials

The following are available online at https://www.mdpi.com/2075-1729/11/3/240/s1.
