# Supplementary material for: Use of Different Types of Biosorbents to Remove Cr (VI) from Aqueous Solution
Source: Life (Basel). 2021 Mar 14;11(3):240. doi: 10.3390/life11030240 (PMC8000416; doi:10.3390/life11030240)
Supplement: Supplementary file 1 [file life-11-00240-s001.pdf]

Supplementary materials for article

"Use of different types of biosorbents to remove Cr (VI) from aqueous solution"

Eva Pertile, Tomáš Dvorský, Vojtěch Václavík and Silvie Heviánková

Although **orange peels** (*Citrus sinensis*) do not belong to the traditional assortment in our geographical latitudes, they have been selected from the set of the tested biosorbents due to their composition (high pectin content). The beneficial properties of pectin that can be used for the sorption of toxic metals have been intensively studied in recent years. One of the prerequisites for the use of pectin-rich biomass is its similarity to alginate, which is a polysaccharide present in algae cells whose previous research has confirmed their high sorption capacity [7]. The carboxyl group of galacturonic acid is the anticipated active site suitable for metal binding. Previous research has been focused mainly on the removal of the following metals: Ni [8–10], Pb [8,9,11–15], As, Cu, Cd [8,9,12], Cu [12,14,16–20], Fe (III), Cr (III) [21,22], Cr (VI) [23–25], Zn (II) [14,15] Co and Mo [10], As [26,27]. The information on the structure and properties of orange peels was taken from the professional literature cited above because the presented findings were practically identical in all works. The use of orange peels as a potential adsorbent material provides a great potential, especially due to their high content of cellulose, pectin, hemicellulose and lignin. The content of pectin in the dry matter of orange peels is within the range of 10-30% [28]. These components contain polar functional groups, (carboxyl and phenolic), which may also be involved in the bond. These are biopolymers that are undoubtedly related to the removal of heavy metals [18]. Orange peel is composed not only of cellulose, pectin, hemicellulose, and lignin, but it also contains chlorophyll pigments and other hydrocarbons with low specific molecular weight [18,28,29]. Perez, Lugo-Lugo et al. [9,18,21,22,29–31] state that the IR spectra show a number of absorption peaks, suggesting the complex nature of the material studied. Since orange peel is mostly composed of cellulose, pectic acid and pectin, O–H, C–O, C=O, C–H, and C–C bonds are expected (see Table 1).

Table 1. Infrared spectrum areas from 4,000 to 400 cm^{-1}, for unmodified orange peels [9].

Functional adsorbent	cm^{-1}	Origin
O–H bond vibration	3,420	cellulose, pectin, hemicellulose absorbed water, and lignin
Symmetric and	2,924	methyl, methoxy and methylene groups
Non-ionic carboxyl	1,744	ester carbonyl (C=O) groups of pectin
Asymmetric vibration	1,638	carboxylate pectin ions (COO^-)
Symmetric vibration	1,434	consequence of aliphatic and aromatic (CH) groups in the plane
COO– symmetric [illegible]	1,267	C–O binding vibrations of carboxylic acids and alcohols
Bond vibrations C–O–C	1,068	

A number of works dealing with the ability of shells of various types of fruit (hazelnuts [32–34], almonds [33,35–37], walnuts [38–43], groundnut [44–47], pistachios [33,48,49], coconuts [50–54], etc.) to sorb metal ions from the aqueous environment can also be found in literature sources. It is cheap, easily available, agricultural waste biomass. Previous research in the field of their possible use for biosorption has focused mainly on the removal of the following metals: Cd, Zn, Cr (III), Cr (VI), Cs+, As and Pb [32,38,39]. Information on the structure and properties of walnut shells (*Juglans regia*) has also been taken from the reference sources mentioned above.

Grain-treated **walnut shells** (*Juglans regia*) are very strong, have a long lifespan and are easily biodegradable. They are not subject to fermentation and are stable over a wide range of temperatures and pH values. Unmodified **walnut shells** (*Juglans regia*) have a relatively complex and multilayer

fibrous lignocellulosic structure. Their surface is rough with a large number of pores, which represent the possible sites for Cr (VI) biosorption by means of physical or chemical mechanism. The main components of walnut shells are cellulose, hemicellulose and lignin, which is the predominant structural component (40.7 – 48.6 %). They also contain some other polar functional groups such as alcohol, carbonyl, carboxyl and phenolic ones [32,38,39,55,57]. The chemical and structural components of the shells are presented in the following Table 2.

Table 2. Structural and chemical composition of walnut shells [56].

Structural component	%
Cellulose	41 – 49
Hemicellulose	25 – 29
Lignin	18 – 27
Chemical composition	%
Ash	0.9 – 1.4
Carbon	46.8 –51.2
Oxygen	44.9
Hydrogen	5.5 – 5.8
Nitrogen	1.4
Potassium	0.51
Phosphorus	0.34
Magnesium	0.22
Sulphur	0.14
Calcium	0.12
Nitrogen	0.10

In the infrared spectrum, there is a Broad band within the range of 3,050 – 3,600 cm^{-1} with a maximum of 3,400 cm^{-1}. We can assign this range to the O–H group (alcohols). Alcohols have a characteristic absorption of the valence vibration of O–H bond within the range of 3,400 – 3,650 cm^{-1}. The exact position of this absorption band depends on the range of hydrogen bonds formed in the molecules. Alcohols bonded by hydrogen bonds have a wider absorption band within the range of 3,300 – 3,400 cm^{-1}, while non-associated alcohols have a rather sharp absorption band around 3,600 cm^{-1}. Alcohols also show an intense absorption band of valence vibration of the C–O bond close to 1,050 cm^{-1} [32,38,39,55,57].

The common occurrence of conifers in the Czech Republic is related to the abundant source of unused **cone biomass** as a renewable resource. The biomass of conifer fruits is itself forest waste and it is essentially a readily available potential biosorbent. The ripe cone consists of epidermis and sclerenchymatic cells, which contain cellulose, hemicellulose and lignin in their cell walls. In addition to that, there are also natural resins and tannins (tanning agents). From a chemical point of view, tanning agents are large polyphenolic compounds that contain hydroxyl and carboxyl groups binding to proteins and other macromolecules [58]. Cone biomass composed of polysaccharides can thus provide binding amino-, carboxyl, phosphate and sulfate groups to the metal-biosorbent bond. The polysaccharide content is about 50 % of the weight in total [59]. A wide range of different types of cones have been tested in the professional literature dealing with biosorption for various metals, such as Pb [60], Cu [58,61–64], Zn [65], Cr (VI) [58,66], Ni (II) [67,68], Cd (II) [60,61,69,70]. The information on the structure and properties of cones was taken from the reference sources mentioned above. The Zeta values of the cone biomass potential were determined at various pH values, including deionized water. Their values were approximately the same, both in deionized water and in the chromium solution. At pH value of 1.0 to 2.0, it was not possible to measure the Zeta potential values due to high ionic strength (the values were slightly positive).

Apricots (*Prunus armeniaca*) and **peaches** (*Prunus persica*), as seasonal fruit, are widely consumed in our geographical latitude. They can also be a serious environmental problem as an agricultural by-product. Apricot and peach stones are therefore an inexpensive and widely available material that can

be further processed for the purpose of biosorption. Alternatively, they can also be a cheap precursor for a source of activated carbon. In professional literature dealing with biosorption, apricot and peach stones have already been studied for the adsorption of e.g. Pb [71], Cd [33], Zn [33], Cu [33,72,73], and Cr (VI) [74]. An analysis of the chemical composition has shown that the main components of stones are again cellulose (30 % of weight) and hemicellulose (28 % of weight), lignin (30 % of weight) and small amounts of lipids (12 % of weight). It has also been found that this agricultural waste contains, together with a high oxygen content (almost 42 % of weight), a relatively large amount of volatile substances (81 % of weight). The ash content was around 0.2 % of the weight [75]. The adsorption band occurring at 3,367 cm^{-1} was assigned to a hydroxyl group, the bands at 2,365 cm^{-1} correspond to NH bond, the peaks within the range from 2,926 cm^{-1} correspond to the $–CH_3$ and $–CH_2$ groups, the peaks around 2,800 – 2,900 cm^{-1} indicate the existence of aldehyde groups, the peak found at 1,579 cm^{-1} was assigned to –CH=CH– and 1,483 cm^{-1} corresponds to –CH bonds [76]. The peak at 1,732 cm^{-1} is assigned to C=O bond in the carboxyl group. This suggests that the functional groups, including carboxyl and hydroxyl groups, will be involved in Cr (VI) adsorption. The surface of the adsorbent was characterized by a large number of pores, which could be, similarly to walnut shells, the sites of potential biosorbent-sorbate bond. The surface area was 861 m^2 g^{-1} [33,76].

Wood-decaying fungus *Fomitopsis pinicola* has been tested to study the biosorption of hexavalent chromium from the aqueous environment. The cell walls of fungi are rigid and provide structural support and shape. They consist of 80 – 90 % of heteropolysaccharides, proteins, lipids, polyphosphates and inorganic ions, which form the walls using sealant mass [77]. Chitin is the common component of the cell wall of fungi. It is strong but flexible. Two layers were found in cell walls of fungi in ultrastructural studies: a thin outer layer consisting of mixed glycans (such as glucans, mannose or galactans) and a thick inner microfibrillar layer of polysaccharide fibres composed of chitin or cellulose with chitin chains in parallel arrangement, sometimes of cellulose chains [5]. Dursun et al. have confirmed the ability of chitin to complex metal ions. Heteropolysaccharides of Fomitopsis have a high content of galactose (heterogalactans) [78]. Numerous enzymes have also been found in Fomitopsis, which are involved in the decomposition of wood. These were mainly enzymes that degraded cellulose [79,80], which they convert into simple sugars [80,81], and lignocellulose [82]. Thermostable xylanase [83] and thermostable cellobiohydrolase [84] have also been isolated from Fomitopsis fruiting bodies. These are enzymes that can be applied in biotechnological processes [85]. The ability of Fomitopsis to degrade polyvinyl alcohol is also interesting, and it could be used for biodegradation of this polymer in various waste materials [86]. Volesky states that various polysaccharides, including cellulose, chitin, alginate, glycan, etc. existing in the cell walls of fungi, play a very important role in metal binding. Some functional groups having the ability to bind metal ions, in particular carboxyl groups, have also been found. There is also evidence confirming that O–, N–, S–, or P– containing groups are directly involved in the bonding of some metals [5]. No works dealing with the tested wood-decaying fungus species have been published in the literature. However, other species such as *Polypores versicolor* (bivalent ions of IIB group) [87], *Phanerochaete chrysosporium*, *Pleurotus ostreatus*, *Trametes versicolor* (bioaccumulation mechanism Cd, Pb and Zn) [88], *Trametes versicolor polyporus* Cr (VI) have been tested [77].

Raw, the so-called **virgin Merino wool**, was used to study the biosorption of Cr (VI). Pure fibre consists of keratin (a proteinaceous substance), pigment and chemically bound water. It is estimated that wool contains more than one hundred and seventy different proteins. However, they are not evenly distributed throughout the fibre. This heterogeneous mixture is responsible for various physical and chemical properties of wool. The proteins They present in wool consist of amino acids (i.e., they contain basic amino groups, $–NH_2$, and acidic carboxyl groups –COOH). The infrared spectrum of pure wool fibres has various characteristic absorption peaks: a broad peak within the range of 3,150 – 3,500 cm^{-1}, which can be connected with –NH– and –SH bonds, as well as strong peaks at 1,630; 1,535 and 1,230 cm^{-1}, belonging to –CONH– (amide I, amide II and C–N extension of amide III, in the specified order). Unmodified wool fibre shows typical differently overlapping tiles on an electron microscope, which appear as edges. Wool fibre consists of three parts, namely cuticle, cortex, and medulla, which was not developed in the used Merino wool. It can be found only in coarse wool [89]. A few studies dealing with this issue have already been published in the professional literature. The authors were dealing with the ability to adsorb, for example, Hg (II), Cu (II) and Co (II) [90], Cr (IV) [91], such as Dakiki et al. [92] who, in addition to other low-cost biosorbents, have also examined the possibility of using wool to remove

Cr (VI). However, since inductively bound plasma spectrometry was used for hexavalent chromium analysis, instead of the commonly used spectrophotometric method with 1,5-diphenylcarbazide, the published results are likely to be biased. This significant shortcoming in the interpretation of data is also pointed out in the review of Miretzky et al. [93].

References:

7. Schiewer, S.; Patil, S.B. Pectin-Rich Fruit Wastes as Biosorbents for Heavy Metal Removal: Equilibrium and Kinetics. *Bioresour. Technol.* **2008**, *99*, 1896–1903, doi:10.1016/j.biortech.2007.03.060.
8. Feng, N.; Guo, X.; Liang, S.; Zhu, Y.; Liu, J. Biosorption of Heavy Metals from Aqueous Solutions by Chemically Modified Orange Peel. *J. Hazard. Mater.* **2011**, *185*, 49–54, doi:10.1016/j.jhazmat.2010.08.114.
9. Liang, S.; Guo, X.; Feng, N.; Tian, Q. Effective Removal of Heavy Metals from Aqueous Solutions by Orange Peel Xanthate. *Trans. Nonferrous Met. Soc. China* **2010**, *20*, s187–s191, doi:10.1016/S1003-6326(10)60037-4.
10. Li, X.; Tang, Y.; Cao, X.; Lu, D.; Luo, F.; Shao, W. Preparation and Evaluation of Orange Peel Cellulose Adsorbents for Effective Removal of Cadmium, Zinc, Cobalt and Nickel. *Colloids Surf. Physicochem. Eng. Asp.* **2008**, *317*, 512–521, doi:10.1016/j.colsurfa.2007.11.031.
11. Shah, J.; Jan, M.R.; ul Haq, A. Removal of Lead From Aqueous Media Using Carbonized and Acid Treated Orange Peel. *Tenside Surfactants Deterg.* **2014**, *51*, 240–246, doi:10.3139/113.110304.
12. Lasheen, M.R.; Ammar, N.S.; Ibrahim, H.S. Adsorption/Desorption of Cd(II), Cu(II) and Pb(II) Using Chemically Modified Orange Peel: Equilibrium and Kinetic Studies. *Solid State Sci.* **2012**, *14*, 202–210, doi:10.1016/j.solidstatesciences.2011.11.029.
13. Lugo-Lugo, V.; Hernández-López, S.; Barrera-Díaz, C.; Ureña-Núñez, F.; Bilyeu, B. A Comparative Study of Natural, Formaldehyde-Treated and Copolymer-Grafted Orange Peel for Pb(II) Adsorption under Batch and Continuous Mode. *J. Hazard. Mater.* **2009**, *161*, 1255–1264, doi:10.1016/j.jhazmat.2008.04.087.
14. Feng, N.; Guo, X. Characterization of Adsorptive Capacity and Mechanisms on Adsorption of Copper, Lead and Zinc by Modified Orange Peel. *Trans. Nonferrous Met. Soc. China* **2012**, *22*, 1224–1231, doi:10.1016/S1003-6326(11)61309-5.
15. Liang, S.; Guo, X.; Tian, Q. Adsorption of Pb2+ and Zn2+ from Aqueous Solutions by Sulfured Orange Peel. *Desalination* **2011**, *275*, 212–216, doi:10.1016/j.desal.2011.03.001.
16. Kumar, K.; Patavardhan, S.S.; Lobo, S.; Gonsalves, R. Equilibrium Study of Dried Orange Peel for Its Efficiency in Removal of Cupric Ions from Water. *Int. J. Phytoremediation* **2018**, *20*, 593–598, doi:10.1080/15226514.2017.1405379.
17. Romero-Cano, L.A.; Gonzalez-Gutierrez, L.V.; Baldenegro-Perez, L.A. Biosorbents Prepared from Orange Peels Using Instant Controlled Pressure Drop for Cu(II) and Phenol Removal. *Ind. Crops Prod.* **2016**, *84*, 344–349, doi:10.1016/j.indcrop.2016.02.027.
18. Feng, N.; Guo, X.; Liang, S. Adsorption Study of Copper (II) by Chemically Modified Orange Peel. *J. Hazard. Mater.* **2009**, *164*, 1286–1292, doi:10.1016/j.jhazmat.2008.09.096.
19. Khormaei, M.; Nasernejad, B.; Edrisi, M.; Eslamzadeh, T. Copper Biosorption from Aqueous Solutions by Sour Orange Residue. *J. Hazard. Mater.* **2007**, *149*, 269–274, doi:10.1016/j.jhazmat.2007.03.074.
20. Feng, N.; Guo, X.; Liang, S. Enhanced Cu(II) Adsorption by Orange Peel Modified with Sodium Hydroxide. *Trans. Nonferrous Met. Soc. China* **2010**, *20*, s146–s152, doi:10.1016/S1003-6326(10)60030-1.
21. Lugo-Lugo, V.; Barrera-Díaz, C.; Ureña-Núñez, F.; Bilyeu, B.; Linares-Hernández, I. Biosorption of Cr(III) and Fe(III) in Single and Binary Systems onto Pretreated Orange Peel. *J. Environ. Manage.* **2012**, *112*, 120–127, doi:10.1016/j.jenvman.2012.07.009.
22. Pérez Marín, A.B.; Aguilar, M.I.; Meseguer, V.F.; Ortuño, J.F.; Sáez, J.; Lloréns, M. Biosorption of Chromium (III) by Orange (Citrus Cinensis) Waste: Batch and Continuous Studies. *Chem. Eng. J.* **2009**, *155*, 199–206, doi:10.1016/j.cej.2009.07.034.
23. Pakshirajan, K.; Worku, A.N.; Acheampong, M.A.; Lubberding, H.J.; Lens, P.N.L. Cr(III) and Cr(VI) Removal from Aqueous Solutions by Cheaply Available Fruit Waste and Algal Biomass. *Appl. Biochem. Biotechnol.* **2013**, *170*, 498–513, doi:10.1007/s12010-013-0202-6.
24. López-Téllez, G.; Barrera-Díaz, C.E.; Balderas-Hernández, P.; Roa-Morales, G.; Bilyeu, B. Removal of Hexavalent Chromium in Aquatic Solutions by Iron Nanoparticles Embedded in Orange Peel Pith. *Chem. Eng. J.* **2011**, *173*, 480–485, doi:10.1016/j.cej.2011.08.018.
25. Pehlivan, E.; Pehlivan, E.; Tutar Kahraman, H. Hexavalent Chromium Removal by Osage Orange. *Food Chem.* **2012**, *133*, 1478–1484, doi:10.1016/j.foodchem.2012.02.037.
26. Ghimire, K.N.; Inoue, K.; Makino, K.; Miyajima, T. Adsorptive Removal of Arsenic Using Orange Juice Residue. *Sep. Sci. Technol.* **2002**, *37*, 2785–2799, doi:10.1081/SS-120005466.
27. Khaskheli, M.I.; Memon, S.Q.; Siyal, A.N.; Khuhawar, M.Y. Use of Orange Peel Waste for Arsenic Remediation of Drinking Water. *Waste Biomass Valorization* **2011**, *2*, 423, doi:10.1007/s12649-011-9081-7.

28. Biswas, B.K.; Inoue, K.; Ghimire, K.N.; Ohta, S.; Harada, H.; Ohto, K.; Kawakita, H. The Adsorption of Phosphate from an Aquatic Environment Using Metal-Loaded Orange Waste. *J. Colloid Interface Sci.* **2007**, *312*, 214–223, doi:10.1016/j.jcis.2007.03.072.
29. Pérez-Marín, A.B.; Zapata, V.M.; Ortuño, J.F.; Aguilar, M.; Sáez, J.; Lloréns, M. Removal of Cadmium from Aqueous Solutions by Adsorption onto Orange Waste. *J. Hazard. Mater.* **2007**, *139*, 122–131, doi:10.1016/j.jhazmat.2006.06.008.
30. Farinella, N.V.; Matos, G.D.; Arruda, M.A.Z. Grape Bagasse as a Potential Biosorbent of Metals in Effluent Treatments. *Bioresour. Technol.* **2007**, *98*, 1940–1946, doi:10.1016/j.biortech.2006.07.043.
31. Li, F.T.; Yang, H.; Zhao, Y.; Xu, R. Novel Modified Pectin for Heavy Metal Adsorption. *Chin. Chem. Lett.* **2007**, *18*, 325–328, doi:10.1016/j.cclet.2007.01.034.
32. Cimino, G.; Passerini, A.; Toscano, G. Removal of Toxic Cations and Cr(VI) from Aqueous Solution by Hazelnut Shell. *Water Res.* **2000**, *34*, 2955–2962, doi:10.1016/S0043-1354(00)00048-8.
33. Kazemipour, M.; Ansari, M.; Tajrobehkar, S.; Majdzadeh, M.; Kermani, H.R. Removal of Lead, Cadmium, Zinc, and Copper from Industrial Wastewater by Carbon Developed from Walnut, Hazelnut, Almond, Pistachio Shell, and Apricot Stone. *J. Hazard. Mater.* **2008**, *150*, 322–327, doi:10.1016/j.jhazmat.2007.04.118.
34. Frenkel, V.S.; Cummings, G.; Maillacheruvu, K.Y.; Tang, W.Z. Food-Processing Wastes. *Water Environ. Res.* **2013**, *85*, 1501–1514, doi:https://doi.org/10.2175/106143013X13698672322462.
35. Bulut, Y.; Tez, Z. Adsorption Studies on Ground Shells of Hazelnut and Almond. *J. Hazard. Mater.* **2007**, *149*, 35–41, doi:10.1016/j.jhazmat.2007.03.044.
36. Demirbas, E.; Kobya, M.; Konukman, A.E.S. Error Analysis of Equilibrium Studies for the Almond Shell Activated Carbon Adsorption of Cr(VI) from Aqueous Solutions. *J. Hazard. Mater.* **2008**, *154*, 787–794, doi:10.1016/j.jhazmat.2007.10.094.
37. Hashemian, S.; Salari, K.; Yazdi, Z.A. Preparation of Activated Carbon from Agricultural Wastes (Almond Shell and Orange Peel) for Adsorption of 2-Pic from Aqueous Solution. *J. Ind. Eng. Chem.* **2014**, *20*, 1892–1900, doi:10.1016/j.jiec.2013.09.009.
38. Pehlivan, E.; Altun, T. Biosorption of Chromium(VI) Ion from Aqueous Solutions Using Walnut, Hazelnut and Almond Shell. *J. Hazard. Mater.* **2008**, *155*, 378–384, doi:10.1016/j.jhazmat.2007.11.071.
39. Wolfová, R.; Pertile, E.; Fečko, P. Removal of Lead from Aqueous Solution by Walnut Shell. *J. Environ. Chem. Ecotoxicol.* **2013**, *5*, 159–167, doi:10.5897/JECE09.025.
40. Hajialigol, S.; Masoum, S. Optimization of Biosorption Potential of Nano Biomass Derived from Walnut Shell for the Removal of Malachite Green from Liquids Solution: Experimental Design Approaches. *J. Mol. Liq.* **2019**, *286*, 110904, doi:10.1016/j.molliq.2019.110904.
41. Lawal, O.S.; Ayanda, O.S.; Rabiu, O.O.; Adebowale, K.O. Application of Black Walnut (Juglans Nigra) Husk for the Removal of Lead (II) Ion from Aqueous Solution. *Water Sci. Technol.* **2017**, *75*, 2454–2464, doi:10.2166/wst.2017.125.
42. Gondhalekar, S.C.; Shukla, S.R. Biosorption of Cadmium Metal Ions on Raw and Chemically Modified Walnut Shells. *Environ. Prog. Sustain. Energy* **2015**, *34*, 1613–1619, doi:https://doi.org/10.1002/ep.12161.
43. Deniz, F. Effective Removal of Maxilon Red Grl from Aqueous Solutions by Walnut Shell: Nonlinear Kinetic and Equilibrium Models. *Environ. Prog. Sustain. Energy* **2014**, *33*, 396–401, doi:https://doi.org/10.1002/ep.11797.
44. Witek-Krowiak, A.; Szafran, R.G.; Modelski, S. Biosorption of Heavy Metals from Aqueous Solutions onto Peanut Shell as a Low-Cost Biosorbent. *Desalination* **2011**, *265*, 126–134, doi:10.1016/j.desal.2010.07.042.
45. Burevska, K.A.; Memedi, H.; Lisichkov, K.; Kuvendziev, S.; Marinkovski, M.; Ruseska, G.; Grozdanov, A. Biosorption of Nickel Ions from Aqueous Solutions by Natural and Modified Peanut Husks: Equilibrium and Kinetics. *Water Environ. J.* **2018**, *32*, 276–284, doi:10.1111/wej.12325.
46. Saha, A.; Bhaduri, D.; Pipariya, A.; Ghosh, R.K. Linear and Nonlinear Sorption Modelling for Adsorption of Atrazine onto Activated Peanut Husk. *Environ. Prog. Sustain. Energy* **2017**, *36*, 348–358, doi:https://doi.org/10.1002/ep.12434.
47. Taşar, Ş.; Kaya, F.; Özer, A. Biosorption of Lead(II) Ions from Aqueous Solution by Peanut Shells: Equilibrium, Thermodynamic and Kinetic Studies. *J. Environ. Chem. Eng.* **2014**, *2*, 1018–1026, doi:10.1016/j.jece.2014.03.015.
48. Moussavi, G.; Barikbin, B. Biosorption of Chromium(VI) from Industrial Wastewater onto Pistachio Hull Waste Biomass. *Chem. Eng. J.* **2010**, *162*, 893–900, doi:10.1016/j.cej.2010.06.032.
49. Moradi, P.; Hayati, S.; Ghahrizadeh, T. Modeling and Optimization of Lead and Cobalt Biosorption from Water with Rafsanjan Pistachio Shell, Using Experiment Based Models of ANN and GP, and the Grey Wolf Optimizer. *Chemom. Intell. Lab. Syst.* **2020**, *202*, 104041, doi:10.1016/j.chemolab.2020.104041.
50. Namasivayam, C.; Sureshkumar, M.V. Removal of Chromium(VI) from Water and Wastewater Using Surfactant Modified Coconut Coir Pith as a Biosorbent. *Bioresour. Technol.* **2008**, *99*, 2218–2225, doi:10.1016/j.biortech.2007.05.023.
51. Pino, G.H.; Souza de Mesquita, L.M.; Torem, M.L.; Saavedra Pinto, G.A. Biosorption of Cadmium by Green Coconut Shell Powder. *Miner. Eng.* **2006**, *19*, 380–387, doi:10.1016/j.mineng.2005.12.003.

52. Costa, A.W.M.C.; Guerhardt, F.; Júnior, S.E.R.R.; Cânovas, G.; Vanale, R.M.; Coelho, D. de F.; Ehrhardt, D.D.; Rosa, J.M.; BasileTambourgi, E.; Santana, J.C.C.; et al. Biosorption of Cr(VI) Using Coconut Fibers from Agro-Industrial Waste Magnetized Using Magnetite Nanoparticles. *Environ. Technol.* **2020**, *0*, 1–12, doi:10.1080/09593330.2020.1752812.
53. do Nascimento, J.M.; de Oliveira, J.D.; Leite, S.G.F. Chemical Characterization of Biomass Flour of the Babassu Coconut Mesocarp (Orbignya Speciosa) during Biosorption Process of Copper Ions. *Environ. Technol. Innov.* **2019**, *16*, 100440, doi:10.1016/j.eti.2019.100440.
54. Gondhalekar, S.C.; Shukla, S.R. Enhanced Adsorption Performance of Oxidised Coconut Coir for Removal of Cd(II) Ions by Multi-Column Arrangement in Series. *Environ. Sci. Pollut. Res.* **2019**, *26*, 28022–28030, doi:10.1007/s11356-019-05995-1.
55. Marshall, W.E.; Champagne, E.T. Agricultural Byproducts as Adsorbents for Metal Ions in Laboratory Prepared Solutions and in Manufacturing Wastewater. *J. Environ. Sci. Health Part Environ. Sci. Eng. Toxicol.* **1995**, *30*, 241–261, doi:10.1080/10934529509376198.
56. Sawalha, M.F.; Peralta-Videa, J.R.; Romero-González, J.; Gardea-Torresdey, J.L. Biosorption of Cd(II), Cr(III), and Cr(VI) by Saltbush (Atriplex Canescens) Biomass: Thermodynamic and Isotherm Studies. *J. Colloid Interface Sci.* **2006**, *300*, 100–104, doi:10.1016/j.jcis.2006.03.029.
57. Wang, X.S.; Li, Z.Z.; Sun, C. Removal of Cr(VI) from Aqueous Solutions by Low-Cost Biosorbents: Marine Macroalgae and Agricultural by-Products. *J. Hazard. Mater.* **2008**, *153*, 1176–1184, doi:10.1016/j.jhazmat.2007.09.079.
58. Ucun, H.; Bayhan, Y.K.; Kaya, Y.; Cakici, A.; Faruk Algur, O. Biosorption of Chromium(VI) from Aqueous Solution by Cone Biomass of Pinus Sylvestris. *Bioresour. Technol.* **2002**, *85*, 155–158, doi:10.1016/S0960-8524(02)00086-X.
59. Micales, J.A.; Han, J.S.; Davis, J.L.; Young, R.A. Chemical Composition and Fungitoxic Activities of Pine Cone Extractives. In *Mycotoxins, Wood Decay, Plant Stress, Biocorrosion, and General Biodeterioration*; Llewellyn, G.C., Dashek, W.V., O'Rear, C.E., Eds.; Biodeterioration Research; Springer US: Boston, MA, 1994; pp. 317–332 ISBN 978-1-4757-9450-2.
60. Kim, N.; Park, M.; Park, D. A New Efficient Forest Biowaste as Biosorbent for Removal of Cationic Heavy Metals. *Bioresour. Technol.* **2015**, *175*, 629–632, doi:10.1016/j.biortech.2014.10.092.
61. Değirmen, G.; Kılıç, M.; Çepelioğullar, Ö.; Pütün, A.E. Removal of Copper(II) and Cadmium(II) Ions from Aqueous Solutions by Biosorption onto Pine Cone. *Water Sci. Technol.* **2012**, *66*, 564–572, doi:10.2166/wst.2012.210.
62. Blázquez, G.; Martín-Lara, M.A.; Dionisio-Ruiz, E.; Tenorio, G.; Calero, M. Copper Biosorption by Pine Cone Shell and Thermal Decomposition Study of the Exhausted Biosorbent. *J. Ind. Eng. Chem.* **2012**, *18*, 1741–1750, doi:10.1016/j.jiec.2012.03.018.
63. Nuhoglu, Y.; Oguz, E. Removal of Copper(II) from Aqueous Solutions by Biosorption on the Cone Biomass of Thuja Orientalis. *Process Biochem.* **2003**, *38*, 1627–1631, doi:10.1016/S0032-9592(03)00055-4.
64. Ofomaja, A.E.; Naidoo, E.B. Biosorption of Copper from Aqueous Solution by Chemically Activated Pine Cone: A Kinetic Study. *Chem. Eng. J.* **2011**, *175*, 260–270, doi:10.1016/j.cej.2011.09.103.
65. Ucun, H.; Aksakal, O.; Yildiz, E. Copper(II) and Zinc(II) Biosorption on Pinus Sylvestris L. *J. Hazard. Mater.* **2009**, *161*, 1040–1045, doi:10.1016/j.jhazmat.2008.04.050.
66. Aoyama, M. Comment on "Biosorption of Chromium(VI) from Aqueous Solution by Cone Biomass of Pinus Sylvestris." *Bioresour. Technol.* **2003**, *89*, 317–318, doi:10.1016/S0960-8524(03)00069-5.
67. Can, M.Y.; Kaya, Y.; Algur, O.F. Response Surface Optimization of the Removal of Nickel from Aqueous Solution by Cone Biomass of Pinus Sylvestris. *Bioresour. Technol.* **2006**, *97*, 1761–1765, doi:10.1016/j.biortech.2005.07.017.
68. Almendros, A.I.; Martín-Lara, M.A.; Ronda, A.; Pérez, A.; Blázquez, G.; Calero, M. Physico-Chemical Characterization of Pine Cone Shell and Its Use as Biosorbent and Fuel. *Bioresour. Technol.* **2015**, *196*, 406–412, doi:10.1016/j.biortech.2015.07.109.
69. Ucun, H.; Bayhana, Y.K.; Kaya, Y.; Cakici, A.; Algur, O.F. Biosorption of Lead (II) from Aqueous Solution by Cone Biomass of Pinus Sylvestris. *Desalination* **2003**, *154*, 233–238, doi:10.1016/S0011-9164(03)80038-3.
70. Ofomaja, A.E.; Naidoo, E.B. Biosorption of Lead(II) onto Pine Cone Powder: Studies on Biosorption Performance and Process Design to Minimize Biosorbent Mass. *Carbohydr. Polym.* **2010**, *82*, 1031–1042, doi:10.1016/j.carbpol.2010.05.024.
71. Tsibranska, I.; Hristova, E. Modelling of Heavy Metal Adsorption into Activated Carbon from Apricot Stones in Fluidized Bed. *Chem. Eng. Process. Process Intensif.* **2010**, *49*, 1122–1127, doi:10.1016/j.cep.2010.08.003.
72. Petrović, M.S.; Šoštarić, T.D.; Pezo, L.L.; Stanković, S.M.; Lačnjevac, Č.M.; Milojković, J.V.; Stojanović, M.D. Usefulness of ANN-Based Model for Copper Removal from Aqueous Solutions Using Agro Industrial Waste Materials. *Chem. Ind. Chem. Eng. Q.* **2015**, *21*, 249–259.

73. Lopičić, Z.R.; Jelena, M.V.; Tatjana, Š.D.; Marija, P.S.; Marija, M.L.; Lačnjevac, Č.; Stojanović, M.D. Influence of PH Value on Cu (II) Biosorption by Lignocellulose Peach Shell Waste Material. *Hem. Ind.* **2013**, *67*, 1007–1015.
74. Bouchalova, M.; Pertile, E.; Surovka, D.; Kučerová, R. Kinetics And Biosorption Efficiency Cr(Vi) By Using Peach Stones (Prunus Persica). In Proceedings of the 13th SGEM GeoConference on Ecology, Economics, Education And Legislation, Albena, Bulgaria, 16-22 June; SGEM World Science: Sofia, Bulgaria, 2013, Volume 1
75. Petrova, B.; Budinova, T.; Tsyntsarski, B.; Kochkodan, V.; Shkavro, Z.; Petrov, N. Removal of Aromatic Hydrocarbons from Water by Activated Carbon from Apricot Stones. *Chem. Eng. J.* **2010**, *165*, 258–264, doi:10.1016/j.cej.2010.09.026.
76. Kobya, M.; Demirbas, E.; Senturk, E.; Ince, M. Adsorption of Heavy Metal Ions from Aqueous Solutions by Activated Carbon Prepared from Apricot Stone. *Bioresour. Technol.* **2005**, *96*, 1518–1521, doi:10.1016/j.biortech.2004.12.005.
77. Venkata Subbaiah, M.; Kalyani, S.; Sankara Reddy, G.; Boddu, V.M.; Krishnaiah, A. Biosorption of Cr(VI) from Aqueous Solutions Using Trametes Versicolor Polyporus Fungi. E-J. Chem. **2008**, 5, 1-12, doi:10.1155/2008/618375.
78. Dursun, A.Y.; Uslu, G.; Tepe, O.; Cuci̇, Y.; Eki̇z, H.İ. A Comparative Investigation on the Bioaccumulation of Heavy Metal Ions by Growing Rhizopus Arrhizus and Aspergillus Niger. *Biochem. Eng. J.* **2003**, *15*, 87–92, doi:10.1016/S1369-703X(02)00187-0.
79. Tsujiyama, S.; Okada, A. Biodegradation of Polyvinyl Alcohol by a Brown-Rot Fungus, Fomitopsis Pinicola. *Biotechnol. Lett.* **2013**, *35*, 1907–1911, doi:10.1007/s10529-013-1281-8.
80. Lee, S.-I.; Kim, J.-S.; Oh, S.-H.; Park, K.-Y.; Lee, H.-G.; Kim, S.-D. Antihyperglycemic Effect of Fomitopsis Pinicola Extracts in Streptozotocin-Induced Diabetic Rats. *J. Med. Food* **2008**, *11*, 518–524, doi:10.1089/jmf.2007.0155.
81. Joo, A.-R.; Jeya, M.; Lee, K.-M.; Sim, W.-I.; Kim, J.-S.; Kim, I.-W.; Kim, Y.-S.; Oh, D.-K.; Gunasekaran, P.; Lee, J.-K. Purification and Characterization of a Beta-1,4-Glucosidase from a Newly Isolated Strain of Fomitopsis Pinicola. *Appl. Microbiol. Biotechnol.* **2009**, *83*, 285–294, doi:10.1007/s00253-009-1861-7.
82. Schilling, J.S.; Tewalt, J.P.; Duncan, S.M. Synergy between Pretreatment Lignocellulose Modifications and Saccharification Efficiency in Two Brown Rot Fungal Systems. *Appl. Microbiol. Biotechnol.* **2009**, *84*, 465–475, doi:10.1007/s00253-009-1979-7.
83. Shin, K.; Jeya, M.; Lee, J.-K.; Kim, Y.-S. Purification and Characterization of a Thermostable Xylanase from Fomitopsis Pinicola. *J. Microbiol. Biotechnol.* **2010**, *20*, 1415–1423, doi:10.4014/jmb.1003.03031.
84. Shin, K.; Kim, Y.H.; Jeya, M.; Lee, J.-K.; Kim, Y.-S. Purification and Characterization of a Thermostable Cellobiohydrolase from Fomitopsis Pinicola. *J. Microbiol. Biotechnol.* **2010**, *20*, 1681–1688.
85. Knežević, A.; Milovanović, I.; Stajić, M.; Lončar, N.; Brčeski, I.; Vukojević, J.; Ćilerdžić, J. Lignin Degradation by Selected Fungal Species. *Bioresour. Technol.* **2013**, *138*, 117–123, doi:10.1016/j.biortech.2013.03.182.
86. Tunali, S.; Kiran, I.; Akar, T. Chromium(VI) Biosorption Characteristics of Neurospora Crassa Fungal Biomass. *Miner. Eng.* **2005**, *18*, 681–689, doi:10.1016/j.mineng.2004.11.002.
87. Şatiroğlu, N.; Yalçınkaya, Y.; Denizli, A.; Arıca, M.Y.; Bektaş, S.; Genç, Ö. Application of NaOH Treated Polyporus Versicolor for Removal of Divalent Ions of Group IIB Elements from Synthetic Wastewater. *Process Biochem.* **2002**, *38*, 65–72, doi:10.1016/S0032-9592(02)00057-2.
88. Baldrian, P. Interactions of Heavy Metals with White-Rot Fungi. *Enzyme Microb. Technol.* **2003**, *32*, 78–91, doi:10.1016/S0141-0229(02)00245-4.
89. Taddei, P.; Monti, P.; Freddi, G.; Arai, T.; Tsukada, M. Binding of Co(II) and Cu(II) Cations to Chemically Modified Wool Fibres: An IR Investigation. *J. Mol. Struct.* **2003**, *650*, 105–113, doi:10.1016/S0022-2860(03)00147-9.
90. Monier, M.; Nawar, N.; Abdel-Latif, D.A. Preparation and Characterization of Chelating Fibers Based on Natural Wool for Removal of Hg(II), Cu(II) and Co(II) Metal Ions from Aqueous Solutions. *J. Hazard. Mater.* **2010**, *184*, 118–125, doi:10.1016/j.jhazmat.2010.08.013.
91. Balkaya, N.; Bektas, N. Chromium(VI) Sorption from Dilute Aqueous Solutions Using Wool. *Desalination Water Treat.* **2009**, *3*, 43–49, doi:10.5004/dwt.2009.473.
92. Dakiky, M.; Khamis, M.; Manassra, A.; Mer'eb, M. Selective Adsorption of Chromium(VI) in Industrial Wastewater Using Low-Cost Abundantly Available Adsorbents. *Adv. Environ. Res.* **2002**, *6*, 533–540, doi:10.1016/S1093-0191(01)00079-X.
93. Miretzky, P.; Cirelli, A.F. Cr(VI) and Cr(III) Removal from Aqueous Solution by Raw and Modified Lignocellulosic Materials: A Review. *J. Hazard. Mater.* **2010**, *180*, 1–19, doi:10.1016/j.jhazmat.2010.04.060.
